# HDAC7 drives glioblastoma to a mesenchymal-like state via LGALS3-mediated crosstalk between cancer cells and macrophages

**DOI:** 10.7150/thno.100939

**Published:** 2024-10-21

**Authors:** Shulin Zhao, Rongrong Zhao, Chuanzheng Wang, Caizhi Ma, Zijie Gao, Boyan Li, Yanhua Qi, Wei Qiu, Ziwen Pan, Shaobo Wang, Qindong Guo, Jiawei Qiu, Yang Fan, Xiaofan Guo, Hao Xue, Lin Deng, Gang Li

**Affiliations:** 1 Department of Neurosurgery, Qilu Hospital, Cheeloo College of Medicine and Institute of Brain and Brain-Inspired Science, Shandong University, Jinan 250012, China.; 2 Shandong Key Laboratory of Brain Health and Function Remodeling, Jinan 250012, China.; 3 Department of Neurosurgery, Provincial Hospital Affiliated to Shandong First Medical University, Jinan, Shandong 250021, China.; 4 Department of Neurosurgery, The First Affiliated Hospital of Shandong First Medical University & Shandong Provincial Qianfoshan Hospital, Jinan, Shandong 250014, China.; 5 Department of Neurology, Loma Linda University Health, Loma Linda 92350, California, USA.

**Keywords:** Glioblastoma, HDAC7, MES transition, LGALS3, M2-like MDMs

## Abstract

**Background:** Glioblastoma multiforme (GBM) is an aggressive brain tumour for which current traditional treatment approaches have been unsuccessful, owing to the high genetic heterogeneity and immunosuppressive tumour microenvironment.

**Methods:** Single-cell and spatial transcriptomic data revealed the niche-specific enrichment of mesenchymal-like (MES-like) GBM cells and monocyte-derived macrophages (MDMs); Gain- and loss-of-function assays of HDAC7 was confirmed both *in vitro* and *in vivo* assays. Mechanistically, mass spectrum, RNA immunoprecipitation (RIP), and co-immunoprecipitation assays were conducted.

**Results:** We found that HDAC7, which upregulated by TRIM28-mediated sumoylation at the protein levels, inhibited SOX8 expression by mediating H3K27 deacetylation. And the down-regulated SOX8 facilitated the transcriptional activity of JUN, to induce LGALS3 secretion, which then bind to the membrane protein ITGB1 on GSC and MDMs in the autocrine and paracrine manners to facilitate the transformation of the mesenchymal phenotype of GBM and the M2 polarization of MDMs, respectively. In turn, LGALS3 could also secreted by M2 MDMs to promote MES transition of GBM in a paracrine manner, creating a positive feedback loop. In translational medicine, we found that blocking LGALS3 improved the therapeutic sensitivity of HDAC inhibitors.

**Conclusions:** Our findings revealed the role of the novel HDAC7-H3K27ac-SOX8/JUN-LGALS3-ITGB1 axis in maintaining the crosstalk between MES GBM and M2 MDM, highlighting that HDAC7 and LGALS3 may serve as potential prognostic biomarkers and therapeutic targets in GBM.

## Introduction

Glioblastoma multiforme (GBM) is an aggressive brain tumour with no effective treatment available. Despite the tremendous efforts of scientists in the field of molecularly targeted therapies over the past decade, clinical trials have all ended in failure 1. Intratumor heterogeneity and cellular dynamic plasticity are hallmarks of glioblastoma, conferring resistance to targeted therapies 2. Recent single-cell RNA sequencing (scRNA) data have indicated that GBM cells could display plasticity within four different states, namely, mesenchymal-like (MES-like), neural progenitor cell-like (NPC-like), astrocyte-like (AC-like), and oligodendrocytic precursor cell-like (OPC-like) states 3, 4. Among them, tumours with mesenchymal-like status have the worst prognosis with significantly increased immune infiltration 5. Understanding the epigenetic characteristics of mesenchymal-like state and origins of its associated transcriptional subtype will be important for elucidating the mechanisms of immune evasion and treatment resistance.

Epigenetic modifications are essential for regulating tumour treatment resistance, and tumour cells develop significant resistance to chemotherapeutic agents through epigenetic changes, particularly aberrant modifications of histones or genomic DNA 6. Therefore, targeting epigenetic modifications is an effective strategy to overcome tumour treatment resistance. Histone deacetylase (HDAC) is involved in transcriptional regulation through the removal of histone acetylation modifications involved in malignant tumour progression 7. HDAC can alter the chromatin state, leading to dysregulation of gene transcription, which can re-express inactivated genes, thereby promoting tumorigenesis 8. Despite the successful clinical experience of HDAC inhibitors, limited efficacy and off-target effects remained urgent issues to be addressed 9. The adaptation of the tumour microenvironment (TME) is a key reason for the inability of current HDAC inhibitors to provide a durable response in tumours 10. The specific combination of epigenetic and microenvironmental cues that confer the different transcriptional states observed in GBM remains poorly understood. Exploring the complex interactions between cancer epigenetics and immunology could provide the basis for new treatment options combining epigenetic drugs and immunotherapy.

Here, we further emphasized the tight localization of MES-like GBM cells and monocyte-derived macrophages (MDMs) and predicted low patient survival by integrating single-cell and spatial transcriptome data 11, 12. We found that HDAC family member HDAC7 was up-regulated by TRIM28-mediated SUMOylation in MES GBM and created a pro-tumour microenvironment by promoting MES transition of GSC and MDM-mediated immunosuppressive transformation. Mechanistically, we found that HDAC7 inhibited the expression of SOX8 through the deacetylation of histone H3K27, and SOX8 interacted with the bZIP domain of JUN to inhibit the transcriptional activity of JUN. LGALS3 regulated by SOX8/JUN complex was secreted by GSC into the tumour microenvironment, which could not only promote GBM MES transition by binding membrane protein ITGB1 on GSC in an autocrine form, but also promote immunosuppression by binding ITGB1 on TAM in a paracrine manner. This work revealed the interdependence of M2-like macrophages in localized regional MES-like tumour cells, resulting in a unique ecological niche of spatial exclusivity. Our findings demonstrated the enhanced therapeutic effect of HDAC inhibitors by disrupting MES-like GBM cell-MDM interactions through LGALS3 inhibitors, providing a combination therapy capable of simultaneously blocking intrinsic tumour cell signalling and remodelling cancer cell mediated immunosuppression. Overall, our results provide a potential therapeutic strategy to target this aggressive and therapy-refractory mesenchymal GBM subtype.

## Materials and Methods

### Ethics statement

This study involving human tissue specimens was authorized by the Ethics Committee of Qilu Hospital of Shandong University. Informed consent was obtained from all individual participants. All animal experiments were approved by the Animal Care and Use Committee of Qilu Hospital, Shandong University and in accordance with the National Guide for the Care and Use of Laboratory Animals.

### Statistical analysis

All results are presented as the mean ± standard error (SEM) and regarded as statistically significant when *P* < 0.05. The results of the statistical analyses were performed with R 4.1.1 and GraphPad Prism 7.00 software. Differences between groups were compared using a one-way ANOVA test or a two-tailed Student's *t* test. The log-rank analysis was used to calculate the significant difference in Kaplan-Meier survival curves.

Description of the experimental procedures are detailed in the Supplementary Materials and Methods.

## Results

### MES-like tumour cells and MDMs form a unique ecological niche for GBM malignant progression

**Figure 1:**
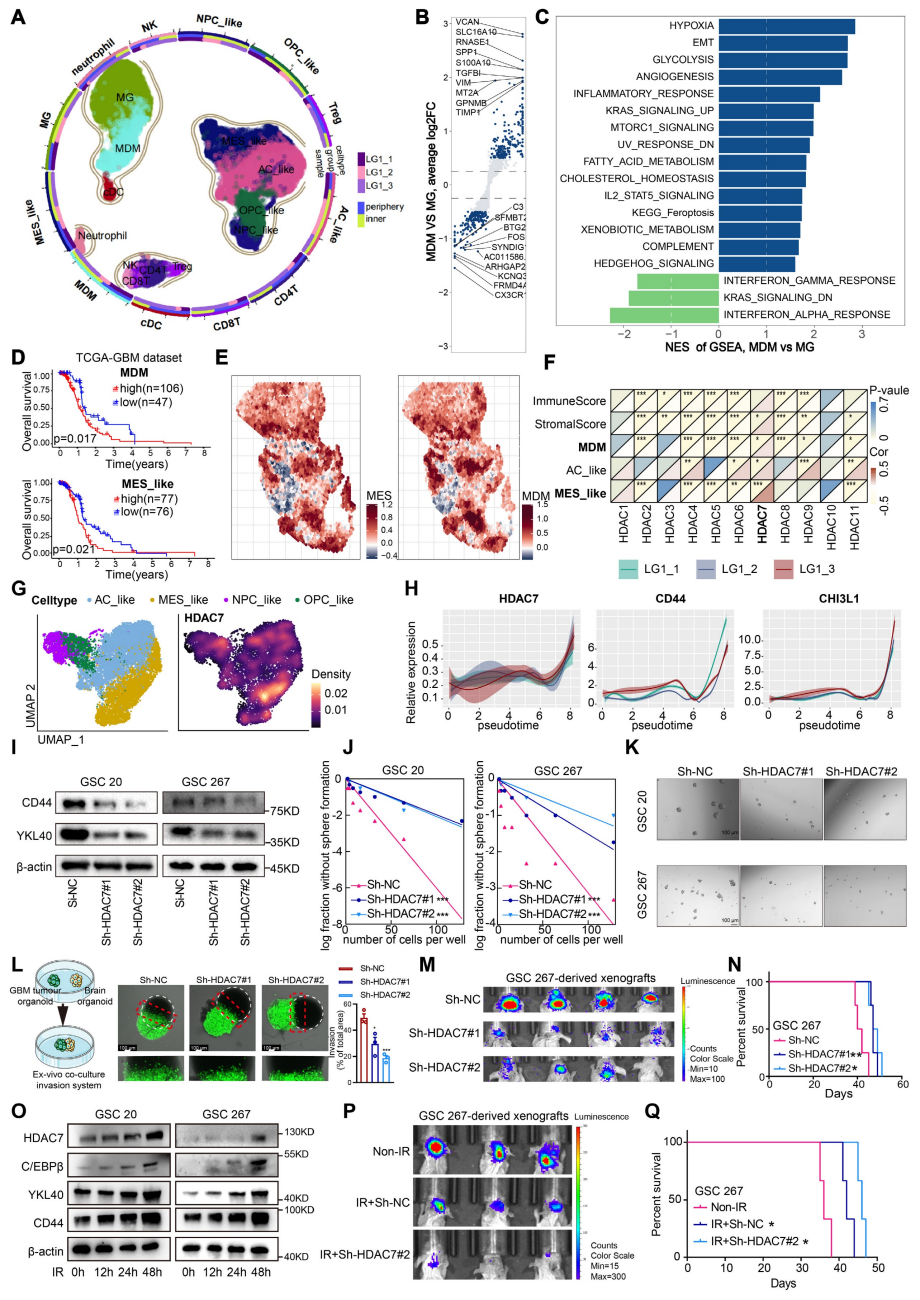
** HDAC7 promotes MES transition and radioresistance of GSCs. A** Umap plot of 29481 cells from normal mucosa and 26185 cells from 3 tumour tissues of 1 GBM IDH^WT^ patient, showing 12 cell types. Each cell type was shown in different colour, and cell type boundaries are outlined by contour curve. R package harmony was used to correct batch effects. **B** Volcano plot showing differential genes between MDMs and MGs. The padj was calculated using Bonferroni correction. **C** Differences in HALLMARK pathway activities scored calculated by GSVA between MDMs and MGs. **D** Kaplan-Meier curves showed overall survival analyses for low and high expression (**up**) MDM and (**down**) MES-like scores in TCGA GBM dataset. **E** Spatial feature plots showing the signature score of (**left**) MES-like GBM cells and (**right**) MDMs in GBM tissue sections. **F** Correlation analysis of HDAC family genes with immuneScore, stromalScore, as well as enrichscore of MDM, AC-like and MES-like GBM cells. **G** Umap and density plots showing HDAC7 was highly expressed in the MES-like GBM subpopulation. **H** Monocle2 pseudotime analysis revealing that the expression of HDAC7 and MES markers (CD44 and CHI3L1) was gradually upregulated with tumour progression. **I** Western blot assays showing the protein expression of CD44 and YKL40 in GSCs transfected with sh-NC or sh-HDAC7. (**J**) Limiting dilution and (**K**) tumour spheres assays were performed for GSCs transfected with sh-NC or sh-HDAC7. Scale bar, 100 µm. **L** *Ex vivo* co-culture invasion assays for GSC tumour spheroid transfected with sh-NC or sh-HDAC7. Scale bar, 100 μm. **M** Bioluminescence image showing the tumour size of mice implanted with luciferase-labelled GSC267 cells expressing sh-HDAC7 or sh-NC. **N** Kaplan-Meier survival curves for mice implanted with luciferase-labelled GSC267 cells expressing sh-HDAC7 or sh-NC. Log-rank analysis was used. **O** Western blot assays showing the protein expression of CD44, YKL40, C/EBPβ and HDAC7 in GSCs at 0, 12, 24 and 48h after IR treatment. **P** Bioluminescence image showing the tumour size of mice implanted with luciferase-labelled GSC267 cells expressing sh-HDAC7 or sh-NC treated with IR or not.** Q** Kaplan-Meier survival curves for mice implanted with luciferase-labelled GSC267 cells expressing sh-HDAC7 or sh-NC treated with IR or not. Log-rank analysis was used. All data are presented as the means ± SEM. The statistical significance is shown as * *P* < 0.05; ** *P* < 0.01; *** *P* < 0.001.

To elucidate the cellular composition of GBM, tumour tissue from three different regions was surgically obtained from a patient with IDH^WT^ GBM and immediately processed for 3′-end single-cell RNA-seq (scRNA) using the 10× Genomics platform. After strict quality control, a total of 26185 cell transcriptomes were retained for subsequent analysis, and twelve major cell clusters with the expression of known marker genes were identified (Figure 1A, Figure S1A, Table S1), including MDMs, Microglia (MGs), Dendritic cells (DCs), Natural Killer cells (NK cells), Neutrophils, CD4+ T cells, Regulatory T cells, CD8+ T cells and four malignant clusters (MES, AC, OPC and NPC), consistent with previous findings that malignant cells exhibited strong intertumoral heterogeneity 3. TAMs, which include MDMs and MGs, were the primary non-tumour infiltrators in the GBM microenvironment (Figure S1B). Furthermore, we analysed the differences and functional enrichment of these two TAM subpopulations and found that the MDM subpopulation was primarily enriched in classical pro-tumour signalling pathways such as hypoxia, EMT and pro-angiogenesis, whereas the MG subpopulation was primarily enriched in interferon-related signalling pathways (Figure 1B, C). Further Kaplan-Meier survival analysis revealed that patients with high enrichment of MES-like GBM cells and MDMs had a worse prognosis (Figure 1D, Figure S1C). Further spatial transcriptome analyses revealed a significant increase of MDM infiltration in the MES-like GBM region (Figure 1E), suggesting that interactive crosstalk between MDM and MES-like GBM cells may drive malignant progression of GBM.

### HDAC7 promotes MES transition and radioresistance of GSCs

To characterise which members of the HDAC family mediate MES transition of GBM and infiltration of MDMs, we performed a transcriptome correlation analysis of the HDAC family in TCGA GBM dataset. Only HDAC7 was positively correlated with MDM infiltration, MES-enriched score (Figure 1F), indicating that HDAC7 is closely associated with MES transition and formation of a suppressive tumour immune microenvironment. Both RNA and protein levels of HDAC7 were significantly increased with higher glioma grade (Figure S2A-C). In addition, survival analysis revealed that GBM patients with high HDAC7 expression had shorter survival duration in several GBM datasets (Figure S2D, E). Furthermore, HDAC7 expression was higher in MES subtype glioma (Figure S2F) and GSCs (Figure S2G). Gene set enrichment analysis (GSEA) also revealed that high HDAC7 expression was strongly enriched in the MES subtype gene set (Figure S2H). Pearson correlation analysis revealed that HDAC7 was positively correlated with MES markers (CD44 and YKL40) and negatively correlated with PN markers (SOX2 and OLIG2) in the TCGA GBM dataset (Figure S2I). As shown by Umap and density plots, HDAC7 was highly expressed in the MES-like GBM subpopulation (Figure 1G). Furthermore, the expression of HDAC7 and MES markers was progressively upregulated with tumour progression (Figure 1H), as demonstrated by Monocle2 pseudotime analysis.

To further confirm the function of HDAC7 in GBM cells, we sought to characterise the changes in cell biological behaviour in HDAC7-knockdown and HDAC7-overexpressing GSCs *in vitro* (Figure S3A, B). Western blot analysis demonstrated that knockdown of HDAC7 suppressed the expression of CD44 and YKL40 (Figure 1I, Figure S3C). Furthermore, neurosphere formation and limiting dilution assays were performed, demonstrating that HDAC7 significantly promoted sphere formation ability (Figure 1J, K, Figure S3D-F). A novel invasion model of GBM tumour spheroids co-cultured with normal rat brain organoids was then established, as previously described 13. In this *ex vivo* model, GBM tumour spheroids with HDAC7 knockdown exhibited reduced invasiveness in rat brain tissue compared to control GBM tumour spheroids (Figure 1L). Conversely, GBM tumour spheroids with HDAC7 overexpression exhibited a stronger invasive capacity (Figure S3G). In addition, *in vivo* experiments revealed that HDAC7 knockdown significantly inhibited tumour growth and prolonged the survival time of tumour-bearing mice (Figure 1M, N, Figure S3H, I). Furthermore, macrophage infiltration assays validated that up-expressed HDAC7 in GBM cells facilitated MDM infiltration, and down-regulated HDAC7 leads to decreased macrophage infiltration (Figure S3J,K).

Radioresistance is a hallmark of MES GSCs 12. GBM patients who received ionizing radiation (IR) treatment in the HDAC7-high group exhibited poorer prognosis in CGGA dataset (Figure S4A). Additionally, the HDAC7-high group exhibited a higher proportion of patients with recurrent glioma than the HDAC7-low group in CGGA dataset (Figure S4B). The results of western blot demonstrated that IR treatment significantly upregulated the expression of MES-related proteins (Figure 1O). Subsequently, the impact of HDAC7 knockdown on the sensitivity of GSCs to radiotherapy was investigated. Following simultaneous application of IR and knockdown of HDAC7, GSCs exhibited significantly higher expression levels of cleaved PARP and γ-H2AX (DNA damage markers) and higher percentages of G2-M phase cells (Figure S4C,D). Furthermore, the combination of HDAC7 knockdown and radiotherapy resulted in a significant reduction in tumour size and an extension of the survival time of tumour-bearing mice (Figure 1P, Q). These results collectively indicated that HDAC7 knockdown significantly inhibits MES transition and radiotherapy resistance in GBM.

### TRIM28-mediated SUMOylation stabilizes HDAC7 protein expression by inhibiting MYCBP2-mediated ubiquitination

**Figure 2:**
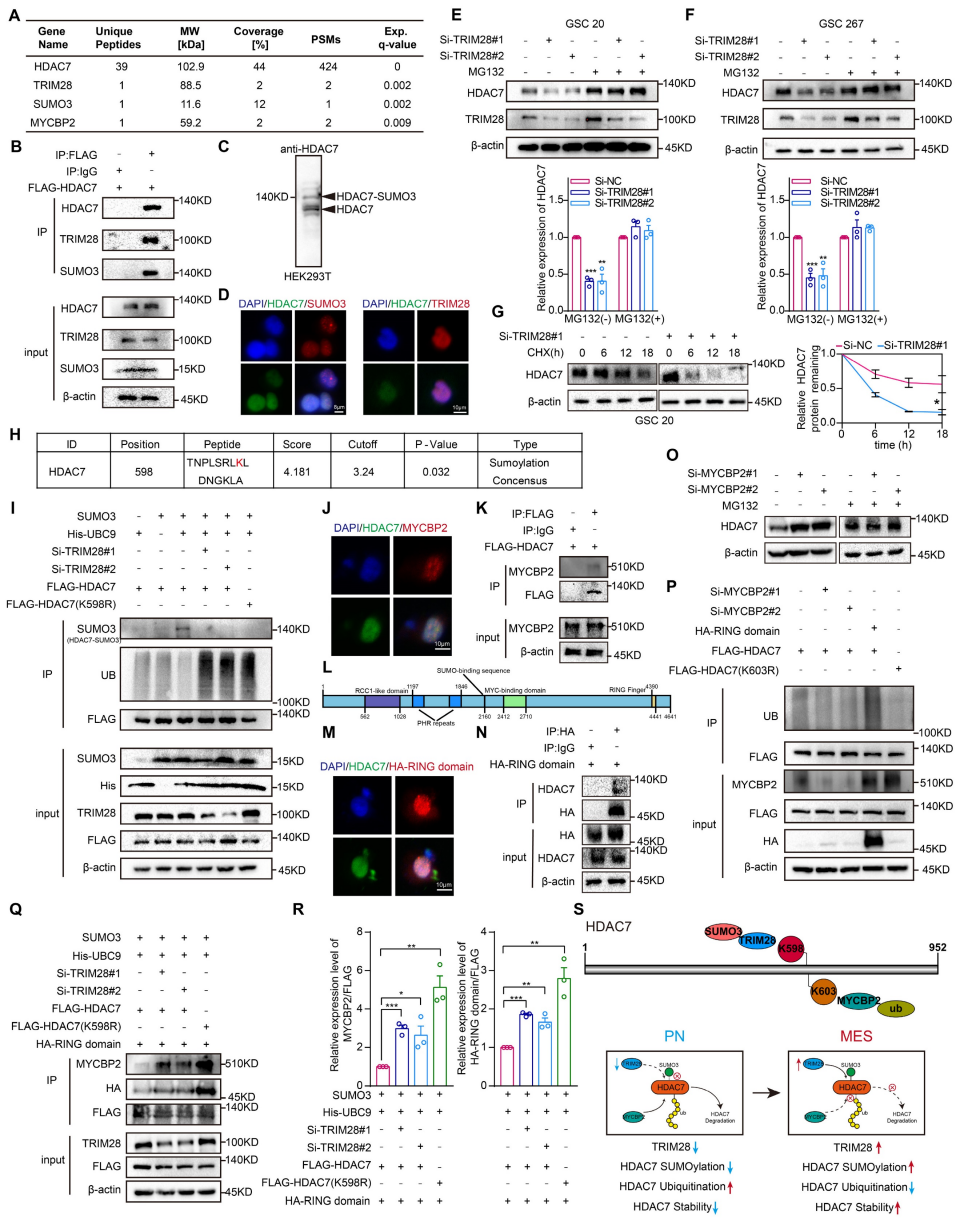
** TRIM28-mediated SUMOylation stabilizes HDAC7 protein expression by inhibiting MYCBP2-mediated ubiquitination. A** Mass spectrometry results of HDAC7 immunoprecipitation showing the interaction of HDAC7 with TRIM28, SUMO3 and MYCBP2. **B** Immunoprecipitation analysis of the association between TRIM28, SUMO3 and HDAC7 in GSC 20 transfected with the indicated plasmids. **C** Western blot assays showing the presence of SUMOylation modification of HDAC7. **D** Immunofluorescence staining experiments showing the colocalization of HDAC7 (green) and TRIM28 (red) or SUMO3 (red) in GSCs. Scale bar, 8 μm (**left**), 10 μm (**right**). Representative western blot assays showing the protein expression level of HDAC7 in TRIM28-knockdown (**E**) GSC20 and (**F**) GSC267 treated with MG132. Quantification histogram showing the protein expression of HDAC7. **G** Western blot assays and a quantitative line graph showing the protein expression level of HDAC7 in TRIM28-knockdown GSCs treated with CHX for the indicated times. **H** GPS-SUMO website (http://sumosp.biocuckoo.org/) predicting the SUMOylation modification site of HDAC7 (K598). **I** Co-IP assays showing the HDAC7 ubiquitination and SUMOylation levels in GSC20 transfected with indicated vectors. **J** Immunofluorescence staining experiments showing the colocalization of HDAC7 (green) and MYCBP2 (red) in GSC20. Scale bar, 10 μm. **K** Co-IP assays showing the interaction of MYCBP2 and HDAC7 in GSC20. **L** Scheme depicting the protein domains of MYCBP2. The numbers refer to the amino acid sequence of full-length human MYCBP2. **M** Immunofluorescence staining experiments showing the colocalization between HDAC7 (green) and RING domain (red) in GSC20. Scale bar, 10 μm. **N** Co-IP assays showing the interaction between the RING domain of MYCBP2 and HDAC7 in GSC20. **O** Western blot assays showing the protein expression level of HDAC7 in MYCBP2-knockdown GSC20 treated with MG132 at the indicated time. **P** Co-IP assays showing the HDAC7 ubiquitination levels in GSC20 transfected with indicated vectors. **Q** Co-IP assays showing the interaction between the RING domain of MYCBP2 and HDAC7 in GSC20 transfected with indicated vectors. **R** Quantification histogram showing the relative expression of MYCBP2/FLAG and HA-RING/FLAG in Figure Q. **S** Schematic representation of the stabilization of HDAC7 protein regulated by SUMOylation (K598) and ubiquitination (K603). All data are presented as the means ± SEM. The statistical significance is shown as * *P* < 0.05; ** *P* < 0.01; *** *P* < 0.001.

The subsequent investigation sought to elucidate the potential mechanisms regulating the expression of HDAC7 protein levels in MES GBM. Liquid chromatography coupled with tandem mass spectrometry identified the E3 SUMO ligases TRIM28 and SUMO3, which played an important role in the specific recognition of target proteins and in the regulation of the activity of the ubiquitin-proteasome degradation system 14-16, as potential HDAC7 interactors (Figure 2A, Table S2), suggesting a possible SUMOylation modification of HDAC7. Co-IP assays confirmed the interaction between HDAC7 with TRIM28 and SUMO3 (Figure 2B). As shown in Figure 2C, we further confirmed the presence of SUMO3 modifications in HDAC7. Subsequent confocal analysis further demonstrated the colocalization of HDAC7 with TRIM28 and SUMO3 (Figure 2D). Furthermore, we observed that the protein level of HDAC7 significantly decreased in TRIM28-knockdown GSCs, which could be reversed by MG132, a selective inhibitor of the proteasome, and protein synthesis inhibitors CHX (Figure 2E-G), indicating that TRIM28 played a pivotal role in regulating the proteasomal degradation of HDAC7 protein.

Subsequently, the GPS-SUMO website was employed to predict the SUMOylation modification region on HDAC7. This analysis demonstrated that K598 may be a potential modification site (Figure 2H, *P* = 0.032). Subsequently, the potential modification site was mutated from lysine (K) to arginine (R) in order to inhibit SUMOylation. The results demonstrated that TRIM28 knockdown resulted in a significant decrease in the SUMOylation modification but a significant increase in the ubiquitination modification of HDAC7, and mutation of the K598 site greatly reduced HDAC7 SUMOylation, whereas the level of ubiquitination was significantly increased, implying that SUMOylation and ubiquitination can be mutually antagonistic on HDAC7 (Figure 2I). Further analysis of the mass spectrometry data revealed that MYCBP2, a key E3 ubiquitin ligase enzyme that mediated substrate recognition in the ubiquitin-proteasome system 17, 18, may also be a potential interactor for HDAC7 (Figure 2A). Subsequent Co-IP and immunofluorescence experiments confirmed the interaction between HDAC7 and MYCBP2 (Figure 2J, K). The RING domain is responsible for the E3 ubiquitin ligase role of MYCBP2 and mediates the ubiquitination modification process of the target protein (Figure 2L) 19, 20. As illustrated in Figure 2M and N, we have demonstrated that HDAC7 indeed bound to the RING domain. Similar to TRIM28, knockdown of MYCBP2 significantly promoted HDAC7 expression, which was reversed by MG132 (Figure 2O). The knockdown of MYCBP2 was found to significantly decrease the ubiquitination of HDAC7, whereas the overexpression of the E3 ubiquitin-linked RING domain significantly increase the ubiquitination of HDAC7 (Figure 2P), suggesting that MYCBP2 was involved in the degradation of ubiquitinated HDAC7. The UbiBrowser, a ubiquitination prediction website, predicted that the K603 site of HDAC7 was a potential ubiquitination site (Figure S5A). To inhibit ubiquitination, the potential modification site was mutated from K to R. The level of ubiquitination of HDAC7 was significantly decreased with the mutation of K603 site (K603R), suggesting that MYCBP2 can function as an E3 ubiquitin-linked enzyme to regulate the protein expression of HDAC7 in a ubiquitin-dependent degradation manner. Further investigation revealed that knockdown of TRIM28 mediated SUMOylation modification reduction resulted in enhanced binding enrichment of MYCBP2 and RING domain to HDAC7 (Figure 2Q, R), which in turn promoted HDAC7 ubiquitination degradation. These results implied that TRIM28-mediated SUMOylation of HDAC7 affected its stability in a ubiquitination-dependent manner by inhibiting the binding of MYCBP2 to HDAC7 (Figure 2S).

### HDAC7 catalyses histone H3K27 deacetylation of SOX8 promoters to inhibit its expression

**Figure 3:**
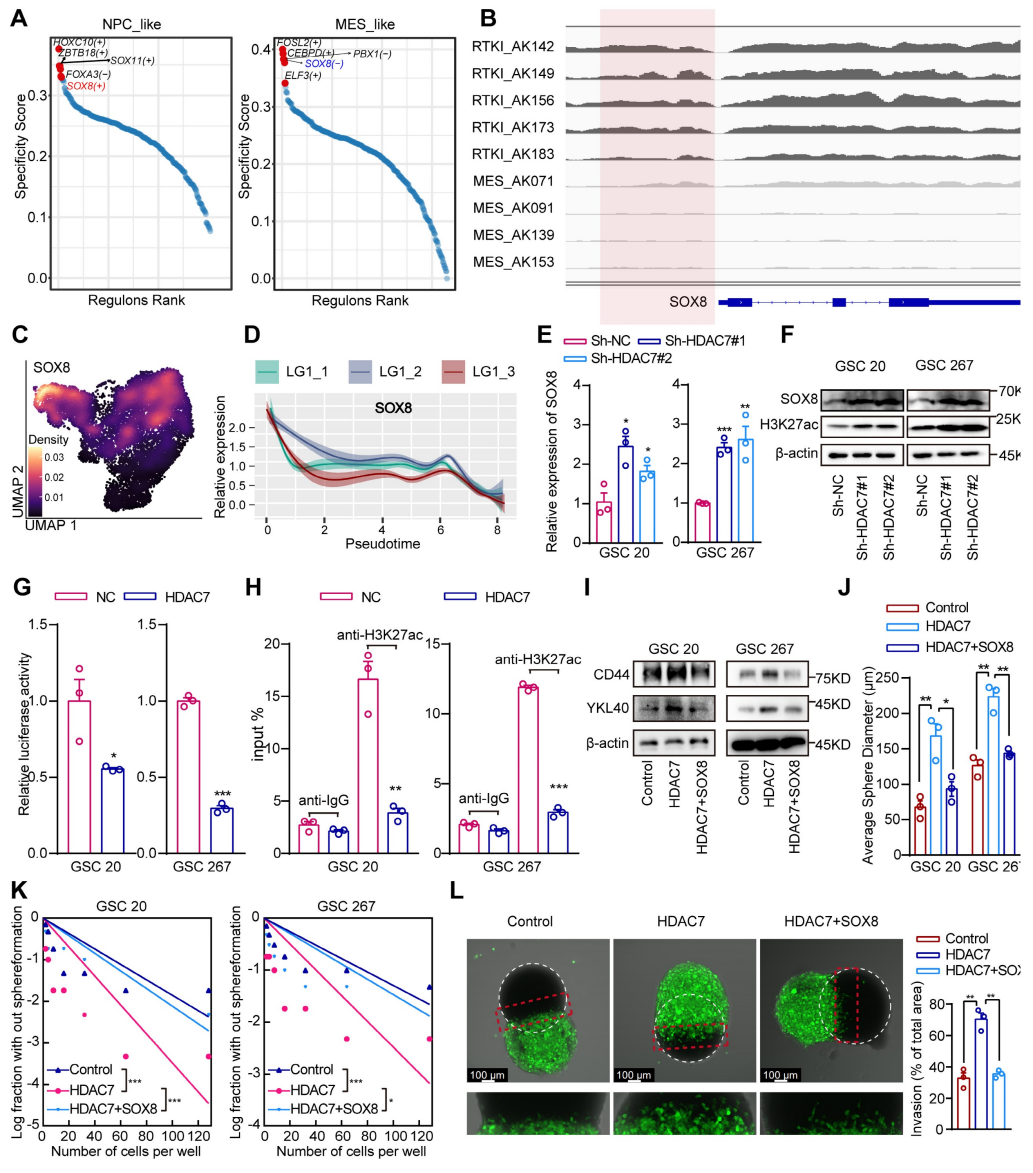
** HDAC7 catalyses histone H3K27 deacetylation of SOX8 promoters to inhibit its expression. A** Rank plots of enriched TFs in (**left**) NPC-like and (**right**) MES-like GBM cells subtypes. **B** IGV software showing the H3K27ac peak distribution on SOX8 promoter in five clinical RTK I and four MES GBM specimens. **C** Density plots showing SOX8 was highly expressed in the NPC-like GBM subpopulation. **D** Monocle2 pseudotime analysis revealing that the expression of SOX8 was gradually downregulated with tumour progression. **E** qRT-PCR assays showing the relative mRNA expression of SOX8 in GSCs transfected with sh-NC or sh-HDAC7. **F** Western blot assays showing the protein expression level of SOX8 and H3K27ac in GSCs transfected with sh-NC or sh-HDAC7. **G** Relative luciferase activity of the SOX8 promoter luciferase reporter in GSCs transfected with ov-NC or ov-HDAC7. **H** CHIP-qPCR assays showing the enrichment of H3K27ac in the promoter of SOX8 in GSCs transfected with ov-NC or ov-HDAC7. **I** Western blot assays showing the protein expression of CD44 and YKL40 in GSCs transfected with ov-NC or ov-HDAC7 and ov-SOX8 as indicated. (**J**) The diameters of the tumour spheres, (**K**) limiting dilution assay, and (**L**) *ex vivo* co-culture invasion assays for GSCs transfected with ov-NC or ov-HDAC7 and ov-SOX8 as indicated. All data are presented as the means ± SEM. The statistical significance is shown as * *P* < 0.05; ** *P* < 0.01; *** *P* < 0.001.

H3K27 acetylation (H3K27ac) is a well-recognised chromatin marker for active enhancers and promoters 21. HDAC7 plays an important role in regulating H3K27ac dynamics 22. Analyses of publicly available ChIP-seq data demonstrated the presence of strong H3K27ac at the promoter region in five clinical RTK I (receptor tyrosine kinase I, commonly with PDGFRA gene amplifications and similar to the PN subtype) and four MES GBM specimens (Figure S6A, B). Cancer master regulators (MRs), including transcription factors (TFs), are proteins that define and regulate tumour cellular states. It has been proposed that systematic identification of cancer MRs would contribute to a better understanding of the heterogeneity of tumour 23, 24. To explore the MRs in different GBM subtypes, we reconstructed scRNA data gene regulatory networks using SCENIC (single-cell regulatory network inference and clustering), a co-expression and motif-based analysis. It was observed that certain TFs exhibited subtype-specific enrichment (Figure S6C), suggesting that TFs regulate genes that are important for subtype identity in GBM. Furthermore, the GBM subtype TFs were ranked and the top 5 active subtype TFs were identified, demonstrating that SOX8 was a positive regulator in the PN GBM and a negative regulator in MES GBM (Figure 3A). Further bioinformatic analysis revealed that H3K27ac was significantly increased in PN GBM samples compared with MES GBM samples in the SOX8 promoter region (Figure 3B). The results of the survival and GSEA analyses indicated that SOX8 was a prognostic protective factor and exhibited a significantly negative correlation with the MES subtype of GBM (Figure S6D, E). The negative correlation between HDAC7 and SOX8 was also supported in the TCGA dataset (Figure S6F). Further analysis of single-cell data indicated that SOX8 was highly expressed in NPC-like GBM cells and was significantly suppressed in MES-like cells (Figure 3C). Furthermore, the expression of SOX8 gradually decreased with tumour progression (Figure 3D). Our transcriptome sequencing analysis also revealed that SOX8 expression was significantly upregulated in HDAC7 knockdown cells (Table S3), which was also confirmed by qPCR assays (Figure 3E). In addition, the protein expression level of SOX8 and global H3K27ac level were significantly increased after knockdown of HDAC7 in GSCs (Figure 3F). Furthermore, overexpression of HDAC7 not only activated HDAC7-mediated repression of SOX8 promoter activity (Figure 3G), but also abrogated the binding of H3K27ac to the SOX8 promoter (Figure 3H). Collectively, these results suggested that HDAC7 represses SOX8 expression by maintaining the deacetylated state of H3K27 on the SOX8 promoter.

Subsequent functional rescue experiments also indicated that HDAC7 overexpression increased the expression of MES markers, sphere-forming ability and invasiveness to rat brain tissue of GSCs, which could be reversed by SOX8 overexpression (Figure 3I-L, Figure S6G). Altogether, our results demonstrated that HDAC7 catalysed H3K27 deacetylation of SOX8 promoter to promote MES transition.

### SOX8 inhibits JUN transcriptional activity by interacting with its bZIP domain

**Figure 4:**
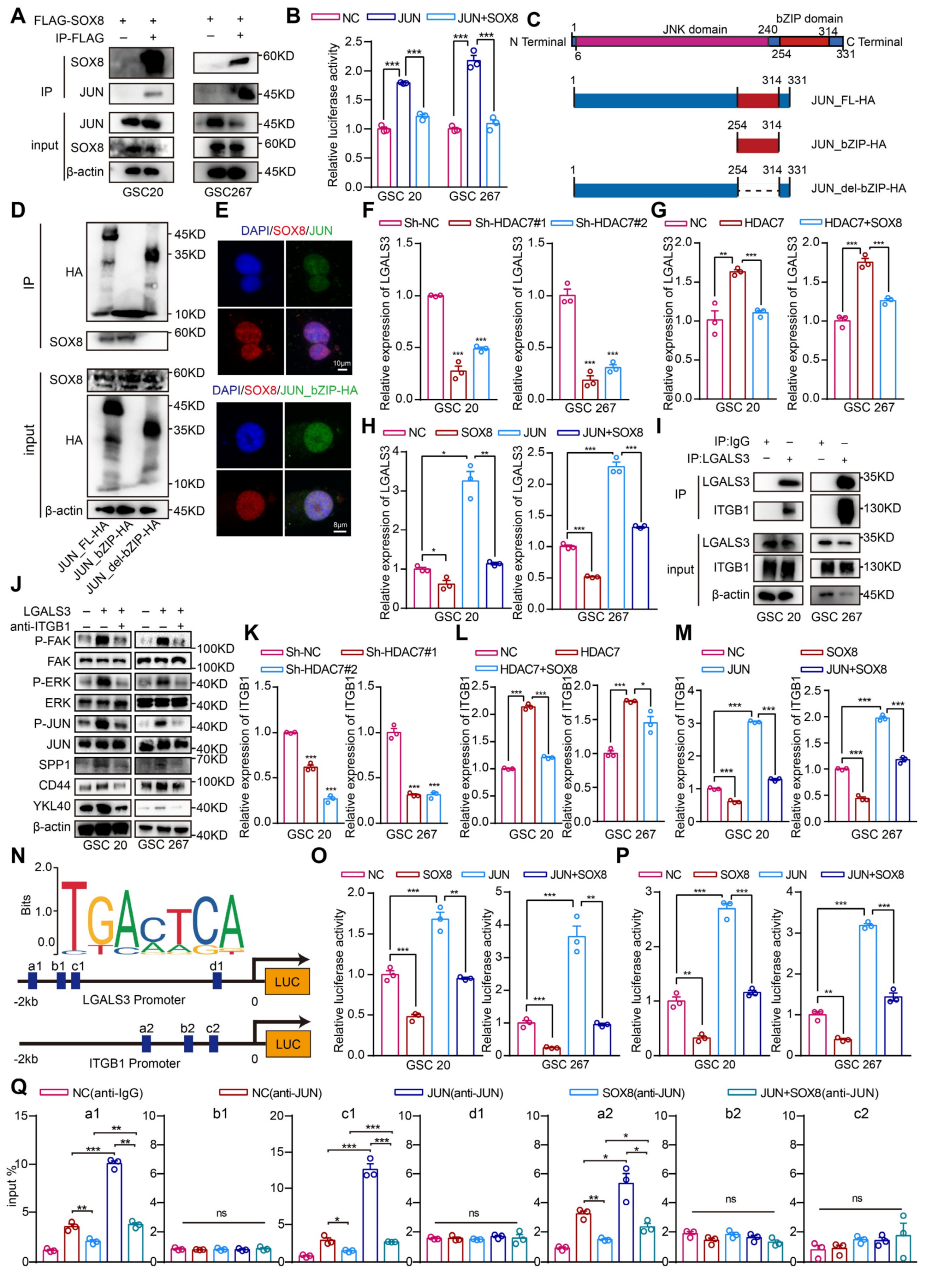
** HDAC7 downregulates SOX8 to promote LGALS3 secretion by GSCs in an autocrine manner to promote MES transition. A** Co-IP assays showing the interaction between the SOX8 and JUN in GSCs. **B** Relative luciferase activity of the JUN binding site luciferase reporter in GSCs transfected with ov-NC, ov-HDAC7 or ov-SOX8 as indicated. **C** Schematic diagram of the structural domain truncated plasmid of JUN. **D** Co-IP assays showing the interaction between the DNA-binding domain of SOX8-bZIP and JUN in GSCs. **E** Immunofluorescence staining experiments showing the colocalisation of SOX8 (red) and JUN (green) or the bZIP domain (green) in GSCs. Scale bar, 10 μm (top), 8 μm (bottom). qRT-PCR assays showing the relative mRNA expression of LGALS3 in GSCs (**F**) transfected with sh-NC or sh-HDAC7, and (**G**) transfected with ov-NC, HDAC7 or SOX8 as indicated, and (**H**) transfected with NC, SOX8, JUN or transfected with SOX8 and JUN as indicated. **I** Co-IP assays showing the interaction between LGALS3 and ITGB1 in GSCs. **J** Western blot assays showing the protein expression of FAK-ERK-JUN signaling pathway and MES phenotype markers (CD44 and YKL40) in GSCs treated with recombinant protein LGALS3 (1 μg/ml, 48h) or blocking antibody anti-ITGB1(5 μg/ml, 48h). qRT-PCR assays showing the relative mRNA expression of LGALS3 in GSCs (**K**) transfected with sh-NC or sh-HDAC7, and (**L**) transfected with NC, HDAC7 or SOX8 as indicated, and (**M**) transfected with NC, SOX8, JUN or transfected with ov-SOX8 and ov-JUN as indicated. **N** Schematic representation of the potential JUN binding loci site on the LGALS3 and ITGB1 promoters. Relative luciferase activity of the (**O**) ITGB1 promoter luciferase reporter, and (**P**) LGALS3 promoter luciferase reporter in GSCs transfected with NC, SOX8, JUN or transfected with SOX8 and JUN as indicated. **Q** CHIP-qPCR assays showing the relative enrichment of JUN in promoters of LGALS3 or ITGB1 in GSCs transfected with NC, SOX8, JUN or transfected with SOX8 and JUN as indicated. All data are presented as the means ± SEM. The statistical significance is shown as *P < 0.05; **P < 0.01; ***P < 0.001.

The enrichment analysis of genes upregulated by HDAC7 revealed that these genes were primarily enriched in signalling pathways such as extracellular matrix organization, cytokine signalling in the immune system, regulation of the MAPK cascade and chemotaxis (Figure S7A, Table S4). Further enrichment analysis in TRRUST demonstrated that the majority of these genes were regulated by the transcription factor JUN (Figure S7B, Table S5), which has been identified as a master regulator of the MES transition 25, 26. Previous studies have demonstrated that the HMG domain of SOX proteins can interact with a variety of transcription factors, including the bZIP protein JUN 27. These interactions have been shown to strongly restrain the promoter activities of JUN, suggesting that SOX8 may regulate downstream functions by repressing the transcriptional activity of JUN. Co-IP assays confirmed the interaction between SOX8 and JUN proteins (Figure 4A). As illustrated in Figure 4B, promoters containing multiple JUN binding sites but no SOX binding sites were effectively activated by JUN in the absence of SOX8, and co-transfection of SOX8 expression plasmids effectively eliminated JUN-dependent reporter gene activation. Moreover, the interaction was found to require specific contacts with the bZIP domain of JUN (Figure 4C-E).

### HDAC7 downregulates SOX8 to promote LGALS3 secretion by GSCs in an autocrine manner to promote MES transition and macrophage infiltration

Analysis of the transcriptomic data revealed that LGALS3 (an important regulator of the tumour microenvironment 28-30), which is a target gene of the transcription factor JUN (Table S5), was the most significantly decreased gene following HDAC7 knockdown (Table S3). LGALS3 has been shown to act as a secreted protein that is typically secreted into microenvironment by tumour and binds to the corresponding receptor to promote malignant tumour progression. Next, it was found that HDAC7 knockdown significantly reduced LGALS3 expression and secretion (Figure 4F, Figure S8A), and the upregulation of LGALS3 expression levels induced by HDAC7 overexpression could be suppressed by SOX8 overexpression (Figure 4G). Meanwhile, SOX8 significantly suppressed the upregulation of LGALS3 expression induced by JUN overexpression (Figure 4H), suggesting that HDAC7 downregulated SOX8 expression and thus activated the transcriptional activity of JUN, which in turn upregulated the expression and secretion of LGALS3.

Integrins play a key role in tumour invasion, matrix remodelling and MAPK signalling pathways 31, 32, which were significantly enriched by HDAC7-upregulated genes (Figure S7A, Table S4). Furthermore, the HitPredict database predicted that LGALS3 may interact with ITGB1, a membrane protein (Figure S8B), which was confirmed by Co-IP experiments and immunofluorescence assays (Figure 4I, Figure S8C), and dozens of pairs of interacting amino acid residues between ITGB1 and LGALS3 were predicted via Alphafold 3 (Figure S8H)^33^. ITGB1 can induce cascade activation of MAPK signalling pathways such as ERK through activation of FAK 34-36, and our scRNA data also revealed that MES-like cells were significantly enriched in signalling pathways such as ERK and AP1 (Figure S8D), suggesting that ITGB1 may be the receptor for autocrine LGALS3 in GSCs. To further confirm whether LGALS3 secreted by GSCs bound to ITGB1 on GSCs in an autocrine form and affected the MES transition, we carried out western blot to detect the activation level of the ITGB1/FAK pathway 35. The result confirmed that the recombinant LGALS3 protein (rLGALS3) activated the FAK/ERK/JUN pathways, which was reversed by an ITGB1-blocking antibody (Figure 4J). Further functional rescue experiments also indicated that rLGALS3 increased the expression of MES markers, sphere-forming ability and invasiveness to rat brain tissue of GSCs, which could be reversed by an ITGB1-blocking antibody (Figure 4J, Figure S8E-G). These results suggested that LGALS3 activated the FAK/ERK/JUN pathway by interacting with the membrane receptor ITGB1 in an autocrine form, thereby promoting the MES transition of GBM.

Similar to LGALS3, we also found that HDAC7 knockdown significantly reduced the expression of ITGB1 (Figure 4K), and the upregulation of ITGB1 expression levels induced by HDAC7 overexpression could be suppressed by SOX8 overexpression (Figure 4L). Meanwhile, SOX8 significantly suppressed the upregulation of ITGB1 expression induced by JUN overexpression (Figure 4M). We therefore hypothesised that the expression levels of both LGALS3 and ITGB1 are regulated by the SOX8/JUN complex. Experiments with luciferase reporter genes confirmed that the SOX8/JUN complex regulated ITGB1 and LGALS3 transcription (Figure 4N-P). To determine the specific sites of action of the SOX8/JUN complex regulating ITGB1 and LGALS3 transcription, we performed ChIP-qPCR experiments, demonstrating that SOX8 affected transcriptional activity by inhibiting the binding of JUN to ITGB1 promoter site a2 and LGALS3 promoter sites a1 and c1, respectively (Figure 4N, Q).

To determine the molecular mechanism underlying the infiltration of MDMs into the MES tumour cell region, we performed differential analysis of MES-like and NPC-like cells in scRNA data. We found that the macrophage chemotactic factor SPP1, which is one of the largest secreted proteins in the tumour microenvironment and can be upregulated by the ERK/JUN signalling pathway 37, was significantly upregulated in MES-like GBM cells (Figure S9A). Subsequent western blot analysis confirmed that rLGALS3 also upregulated the expression of SPP1, which was reversed by an ITGB1-blocking antibody (Figure 4J). The LGALS3/JUN/ITGB1/SPP1 genes were highly expressed in MES-like GBM cells (Figure S9B), and the expression of these genes was significantly upregulated with tumour progression (Figure S9C). Further spatial transcriptome data demonstrated the spatial exclusivity of SOX8 expression with the expression of JUN, CD44, SPP1 and LGALS3 (Figure S9D). Together, these results indicated that HDAC7 downregulated SOX8 to promote GBM MES transition and macrophage infiltration via the LGALS3-ITGB1 autocrine axis.

### LGALS3-ITGB1 mediates the crosstalk between MES GBM and M2 TAM

**Figure 5:**
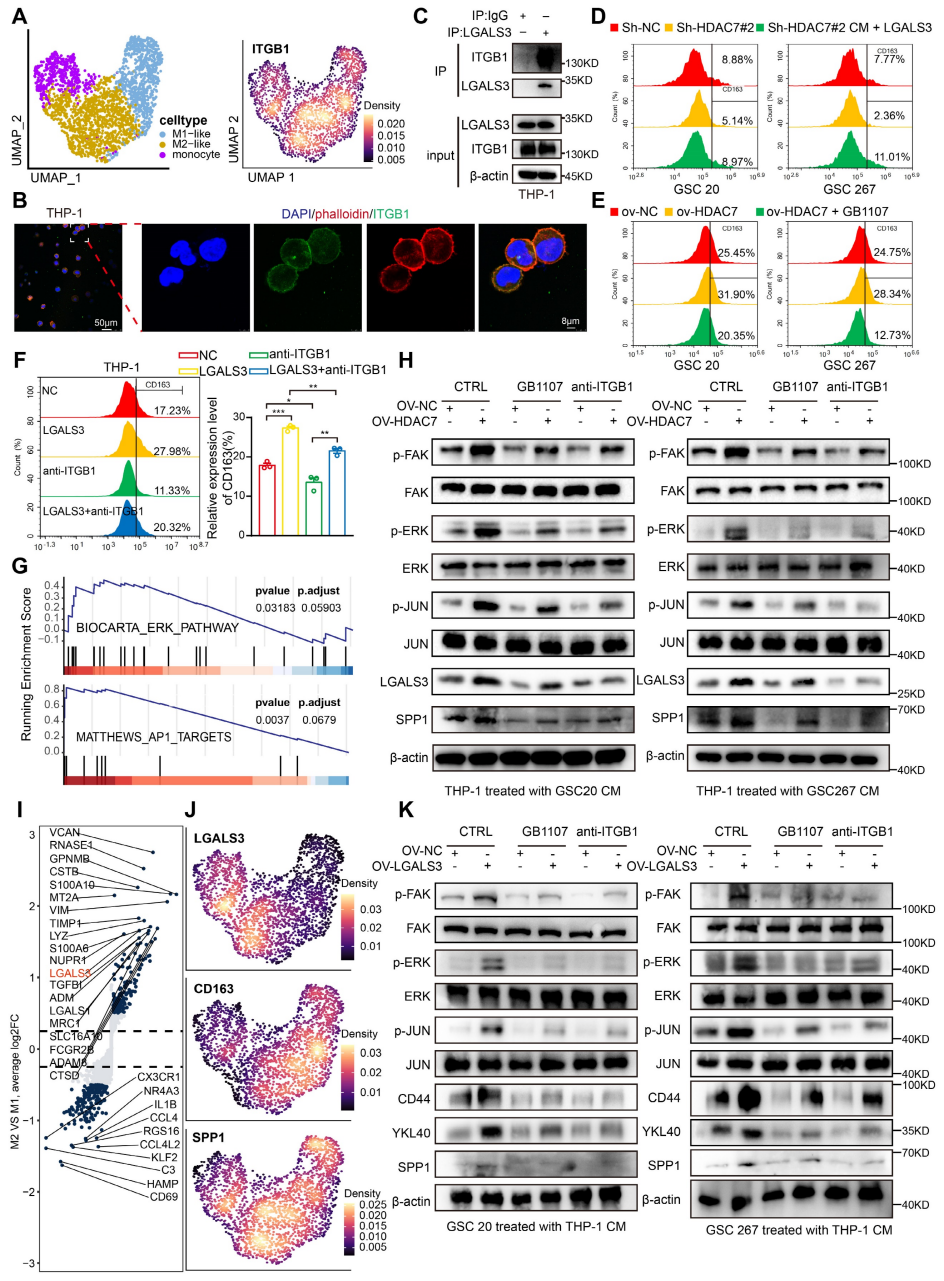
** LGALS3-ITGB1 mediates the crosstalk between MES GBM and M2 TAM. A** Umap and density plots showing that ITGB1 was highly expressed in the M2-like MDM subpopulation. **B** Immunofluorescence staining experiments showing the colocalisation of phalloidin (red) and ITGB1 (green) in THP-1, revealing that ITGB1 is mainly present in the cell membrane. Scale bar, 50 μm (left), 8 μm (right). **C** Co-IP assays showing the interaction between LGALS3 and ITGB1 in macrophages. Flow cytometry assay showing the proportion of CD163+ in THP1 differentiated macrophages treated with CM collected from GSCs (**D**) transfected with sh-NC, sh-HDAC7 or cotreated with rLGALS3, (**E**) transfected with NC, HDAC7 or co-treated with GB1107, and (**F**) treated with NC, rLGALS3 (1 μg/ml, 48h), blocking antibody anti-ITGB1 (5 μg/ml, 48h) or co-treated with rLGALS3 (1 μg/ml, 48h) and anti-ITGB1 (5 μg/ml, 48h). **G** GSEA analysis indicating that M2-like cells were significantly enriched in ERK and AP1 signalling pathway. **H** Western blot assays showing the protein expression of LGALS3, SPP1 and FAK-ERK-JUN signalling pathway in THP-1 differentiated macrophages cotreated with CM from GSCs treated with GB1107 or anti-ITGB1 as indicated. **I** Volcano plot showing differential genes between M2-like and M1-like MDMs. Padj was performed with Bonferroni correction. **J** Density plots showing LGALS3, CD163 and SPP1 was highly expressed in the M2-like MDM subpopulation. **K** Western blot assays showing the protein expression of CD44, YKL40, SPP1 and FAK-ERK-JUN signalling pathway in GSCs co-treated with CM from THP-1 differentiated macrophages treated with NC, LGALS3, GB1107 or anti-ITGB1 as indicated. All data are presented as the means ± SEM. The statistical significance is shown as *P < 0.05; **P < 0.01; ***P < 0.001.

We further explored the MDMs and annotated three subpopulations (monocytes, M1-like and M2-like subpopulations) based on marker genes (Figure 5A, Figure S10A). Further GSVA functional enrichment analysis revealed that M2-like macrophages were mainly enriched in tumour-promoting pathways, such as EMT and angiogenesis (Figure S10B). As our data suggested that ITGB1 is the key receptor for LGALS3-mediated signalling in GSCs, we next examined whether ITGB1 is also expressed on the cell membrane of TAMs. As shown in Figure 5A and B, we found that the membrane protein ITGB1 was highly expressed in MDMs, especially in M2-like cells. Co-IP experiments also confirmed that LGALS3 and ITGB1 can also interact with each other in macrophages (Figure 5C).

To further demonstrate that HDAC7 is responsible for M2 polarization of TAMs via the LGALS3 pathway, we collected supernatants from the NC and HDAC7 knockdown groups to treat THP1-differentiated macrophages. Our qRT-PCR analysis data revealed that HDAC7 knockdown significantly inhibited the expression of M2 macrophage markers, including CD206, CD163, IL-10, ARG1 and TGF-β (Figure S10C, D). Flow cytometry results further demonstrated that compared with conditioned medium (CM) from the NC group, CD163 was significantly downregulated in THP-1 cells treated with CM derived from GSCs with HDAC7 knockdown, which could be rescued by rLGALS3 (Figure 5D, Figure S10E). Meanwhile, treatment of THP-1 cells with CM derived from GSCs overexpressing HDAC7 significantly increased CD163 levels, which could be reversed by the LGALS3 inhibitor GB1107 (Figure 5E, Figure S10F). rLGALS3 significantly increased the level of CD163 expression, whereas anti-ITGB1 inhibited CD163 expression (Figure 5F). Further GSEA of our scRNA data also indicated that M2-like cells were significantly enriched in signalling pathways such as ERK and AP1 (Figure 5G). Subsequent western blot assays confirmed that treatment of THP-1 cells with CM derived from GSCs overexpressing HDAC7 significantly activated the FAK-ERK-JUN pathway, which could be reversed by the LGALS3 inhibitor GB1107 and anti-ITGB1 (Figure 5H). In summary, we demonstrated that LGALS3 secreted by GSCs binds to the ITGB1 membrane receptor of TAMs in a paracrine secretion and activates the FAK-ERK-JUN pathway to promote M2 polarization of TAMs.

Further single-cell data analysis revealed that in addition to CD163, the LGALS3 and SPP1 genes were also highly expressed in M2 macrophages (Figure 5I, J). We also confirmed that treatment of THP-1 cells with CM derived from GSCs overexpressing HDAC7 significantly upregulated the expression of LGALS3 by FAK-ERK-JUN pathway, which could be reversed by the LGALS3 inhibitor GB1107 and anti-ITGB1 (Figure 5H). Therefore, we sought to investigate whether upregulated LGALS3 in TAMs could influence MES transition of GBM in a paracrine manner. We then treated GSCs with CM derived from THP1 overexpressing LGALS3, and the results revealed that the FAK-ERK-JUN signalling pathway, SPP1 and MES markers were also upregulated in GSCs (Figure 5K). These results demonstrated that LGALS3, which was up-regulated by the FAK-ERK-JUN pathway, can be secreted by the M2 TAM to promote MES transition of GBM in a paracrine form.

In conclusion, we have shown that LGALS3-ITGB1 mediates the interaction between MES GBM and M2 TAM, which together drive malignant tumour progression.

### Combination of HDAC and LGALS3 inhibitors suppresses GBM malignant progression

**Figure 6:**
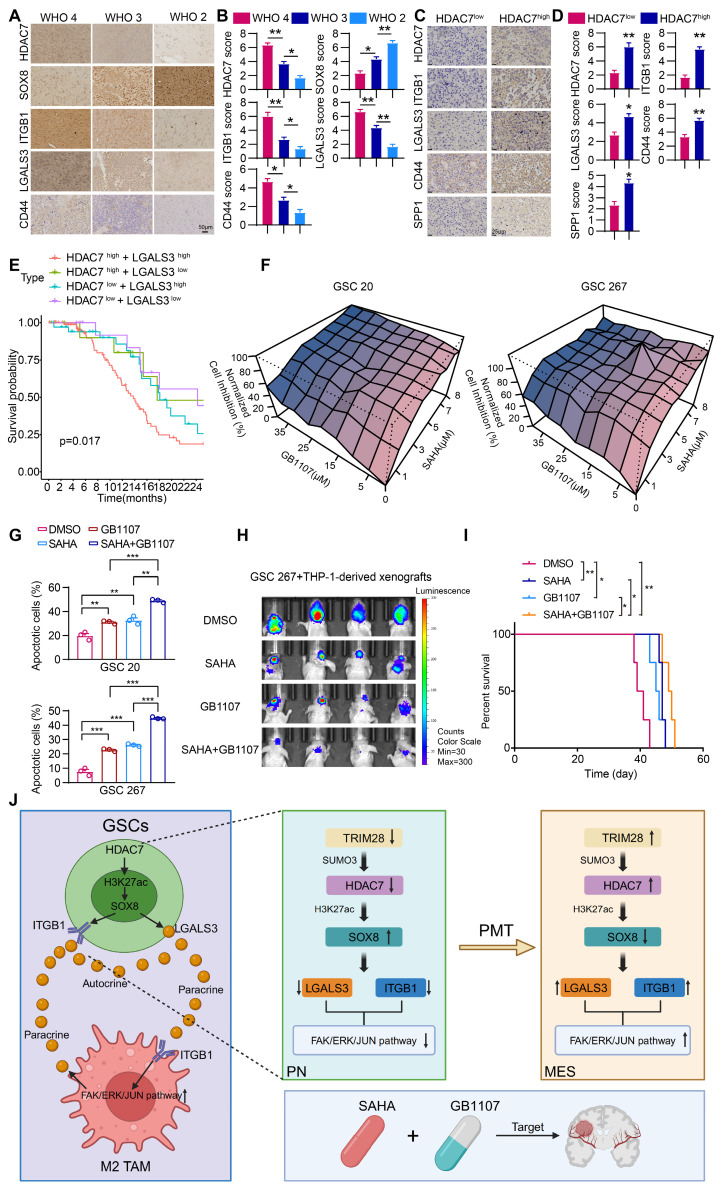
** Combination of SAHA and GB1107 inhibits GBM progression.** (**A**) Representative IHC-staining images and (**B**) quantifcation histogram showing the relative expression of HDAC7, SOX8, ITGB1, LGALS3 and CD44 in different WHO grade glioma samples. Scale bar, 50 μm. (**C**) Representative IHC-staining images and (**D**) quantifcation histogram showing the relative expression of HDAC7, ITGB1, LGALS3, SPP1 and CD44 in HDAC7^high^ and HDAC7^low^ GBM samples. Scale bar, 25 μm. **E** Kaplan-Meier curves showed overall survival analyses for low or high expression HDAC7 and LGALS3 in TCGA GBM dataset. **F** The normalized cell inhibition (%) in GSCs treated with different concentrations of SAHA and GB1107 for 48 h. **G** Apoptotic cell analysis in GSCs treated with SAHA (5μm) and GB1107 (20μm) for 48 h. **H** Bioluminescence image showing the tumor size of mice orthotopically implanted with luciferase-labeled GSC267 and THP1 differentiated macrophages treated with SAHA (20 mg/kg) or GB1107 (20 mg/kg) for the indicated times. **I** Kaplan-Meier survival curves for mice implanted with and THP1 differentiated macrophages treated with SAHA (20 mg/kg) or GB1107 (20 mg/kg). Log-rank analysis was used, n = 4 for each group. **J** A schematic model showing the mechanisms of HDAC7 playing an essential role in maintaining MES phenotype and promoting M2-like polarization of TAMs. Image was created in BioRender, with permission. All data are presented as the means ± SEM. The statistical significance is shown as * *P* < 0.05; ** *P* < 0.01; *** *P* < 0.001.

Our study showed that space is created within the tumour over time, leading to a highly heterogeneous tumour microenvironment that generates spatially adaptive ecological niches, making the efficacy of monotherapy very limited. The clinical relevance of our experimental findings was supported by immunohistochemical staining analyses demonstrating that the expression of HDAC7, ITGB1, LGALS3 and CD44 was highest in GBM tumours and was negatively correlated with SOX8 expression (Figure 6A, B). The expression of ITGB1, LGALS3, SPP1 and CD44 was higher in HDAC7-high GBM tissues compared to HDAC7-low tissues (Figure 6C, D), which were consistent with our conclusion.

There is some evidence that epigenetic drugs may also play an important role in synergy with other anticancer therapies or in reversing acquired treatment resistance 38. We found that the secreted factor LGALS3 played a key role in establishing the positive feedback loop between cancer cells and MDMs, which may be an attractive therapeutic target. Thus, blocking the MDM-mediated immunosuppressive microenvironment with the LGALS3 inhibitor GB1107 in combination with the HDAC7 inhibitor SAHA may be an effective way to treat GBM. Survival analyses in the TCGA database showed that high expression of HDAC7 combined with high expression of LGALS3 exhibited the shortest survival time (Figure 6E). *In vitro* experiments demonstrated a significant synergistic inhibitory effect of GB1107 and SAHA on the malignant progression of GSC (Figure 6F, G). The exciting *in vitro* results drove us to further establish a mouse model of GBM *in situ* by co-injection of GSCs with macrophages to explore the efficacy of the combination therapy *in vivo*. The combination therapy effectively inhibited GBM tumour growth, significantly reduced tumour volume, and significantly prolonged the survival of mice (Figure 6H, I). In conclusion, we have developed a combination therapy that simultaneously inhibits intrinsic tumour cell signalling and reprograms the immunosuppressive microenvironment to improve the therapeutic efficacy of mesenchymal GBM.

## Discussion

The adaptability of the tumour microenvironment is a key reason that current targeted anti-tumour therapies fail to achieve durable responses in GBM. Recently, scRNA-seq has been used to classify malignant cells in GBM were classified into four potentially plastic cell states. Among these, the MES-like state exhibits increased immune macrophage infiltration. In this work, we further emphasised the tight localisation of MES-like GBM cells and MDMs by integrating single-cell and spatial transcriptome data. We found that in MES GBM cells, HDAC7, a member of the HDAC family, is regulated by SUMOylation and is upregulated. We found that HDAC7 facilitated a pro-tumour microenvironment by promoting the malignancy of GSCs and immunosuppressive transformation of MDMs by catalysing histone H3K27 deacetylation of SOX8 promoters to inhibit its expression. SOX8 inhibited JUN transcriptional activity by interacting with its bZIP domain, which induced LGALS3 secretion, thereby activating ITGB1-FAK-ERK-JUN signalling to facilitate GBM MES transition in an autocrine mechanism and a highly immunosuppressive TME in a paracrine manner. This work revealed the interdependence of M2-like macrophages in localized regional MES-like tumour cells, resulting in a unique ecological niche of spatial exclusivity (Figure 6J). Our findings improved HDAC inhibitor therapy by disrupting MES-like and M2-like MDM interactions with LGALS3 inhibitors, providing a potential therapeutic strategy.

HDAC7 has been demonstrated to play an important role in regulating malignant progression in a variety of tumours recently 39-41. However, little is known about its role in mediating crosstalk between tumour cells and TAMs. We identified HDAC7 as a key epigenetic regulator of MES transition and GSC-TAM crosstalk in GBM. We demonstrated that knockdown of HDAC7 in GSCs not only inhibited GBM PN to MES transition but also greatly increased the sensitivity of cells to radiotherapy (Figure 1). TFs are proteins that define and regulate tumour cellular states 23. Previous studies have identified SOX10 as a particularly interesting candidate to study epigenetic control and remodelling of subtype gene regulation in GBM 42, and found that SOX8 has the ability to trigger the process of ferroptosis via altering glycolipid and iron metabolism in hepatocellular carcinoma 43. However, the exact mechanism of SOX8 in glioma is still unknown 44. In our study, we found that SOX8, another SOX gene family member, is also a master positive regulator of PN-like GBM and a negative regulator of MES-like GBM, with both tumour cell intrinsic and microenvironmental effects. Meanwhile, SOX8 had a lower H3K27ac signal in the MES-like GBM (Figure 3B), suggesting that SOX8 may be a potential target for epigenetic control and subtype gene regulatory remodelling in glioblastoma.

We found that SOX8 maintained the PN subtype of the GBM state and that its loss caused a shift to a mesenchymal phenotype. To explore the specific molecular mechanisms downstream of SOX8 regulation of the transcriptomic state of GBM, we analysed the HDAC7-mediated transcriptomic gene regulatory network and found that most of the genes regulated by HDAC7 are targets of the transcription factor JUN (Figure S7B). SOX proteins are widely believed to team up with other transcription factors as partner proteins to perform their many essential functions during development, especially SOX8 and SOX10 27. In our work, we demonstrated that SOX8 can bind to the bZIP structural domain of the JUN transcription factor to influence the efficient activation of its target genes (Figure 4), highlighting that this flexibility in selecting interaction partners may be one of the reasons for the pleiotropic function of the SOX8 protein.

Today, therapies targeting epigenetic dysregulation appear to be promising adjuncts to other cancer therapies. However, the clinical outcomes of combination therapies containing epigenetic agents have been disappointing due to their limited efficacy 38. Combination therapies have always been explored to combat drug resistance in cancer and have spawned a range of practice-changing combinations. Dual blockade of oncogenic signalling by inhibiting multiple nodes of a pathway, either downstream of the driver oncogene or upstream of the driver cancer-based oncogene, has proven to be a reasonably effective way to prolong the inhibitory response to oncogenic pathways 45. In this work, we demonstrated that downstream of oncogenic HDAC7, LGALS3-ITGB1-mediated activation of the FAK-ERK-JUN pathway played a key role in the MES transition of GBM and the formation of a TAM-dependent suppressive TME (Figure 5), suggesting that LGALS3 was a potentially effective candidate for a therapeutic strategy that has the potential to exhibit good clinical results from both tumour suppression and optimization of HDAC inhibitors. Consistent with our expectations, extensive experiments showed that the combination of an HDAC inhibitor and an LGALS3 inhibitor had a significant synergistic effect in the treatment of MES GBM, which was able to simultaneously block HDAC7-driven intrinsic tumour cell signalling and re-programme the suppressive TME. This work provides new ideas and avenues for the treatment of the highly aggressive and refractory mesenchymal subtype of GBM.

## Supplementary Material

Supplementary materials and methods, figures and tables 6-7.

Supplementary tables 1-5.
