# Supplementary material for: HDAC7 drives glioblastoma to a mesenchymal-like state via LGALS3-mediated crosstalk between cancer cells and macrophages
Source: Theranostics. 2024 Oct 21;14(18):7072–87. doi: 10.7150/thno.100939 (PMC11610139; doi:10.7150/thno.100939)
Supplement: Supplementary file 1 — Supplementary materials and methods, figures and tables 6-7. [file thnov14p7072s1.pdf]

Supplementary Figure 1

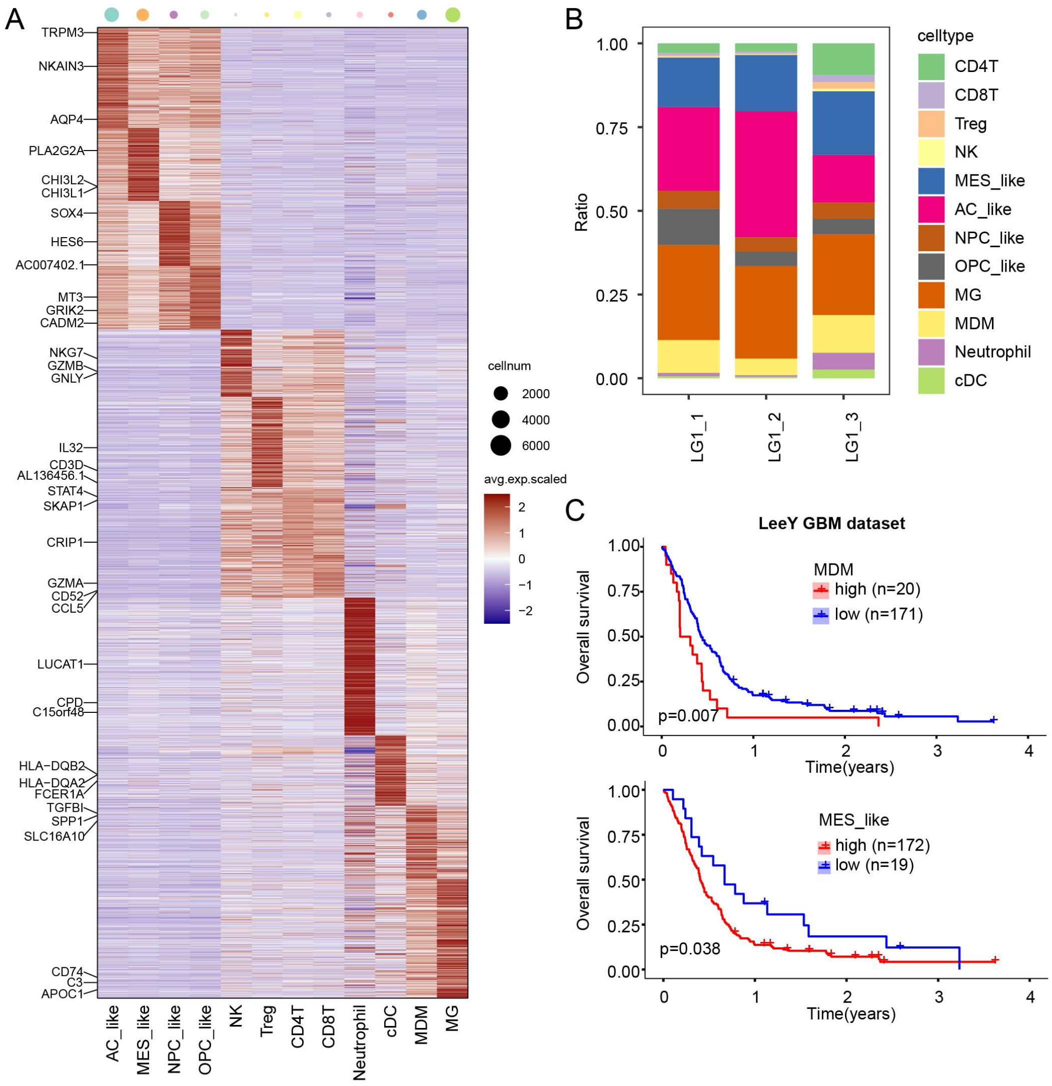

Supplementary Figure 1. MES-like GBM cells and MDMs form a unique ecological niche for the progression of malignant tumour.

A In the heat map of 29,481 cells from normal mucosa and 26,185 cells from 3 tumour tissues of 1 GBM IDH^{WT} patient, 12 main cell clusters with the expression of known marker genes were shown. Each cell type is shown in a different colour and the number of cells is represented by the size of the circle. **B** Analysis of the percentage of 12 cell types from 3 tumor tissues of 1 GBM IDH^{WT} patient in the GBM microenvironment. **C** Kaplan-Meier curves showing overall survival analyses for low and high expression (**up**) MDM and (**down)** MES-like scores in the LeeY GBM dataset.

Supplementary Figure 2

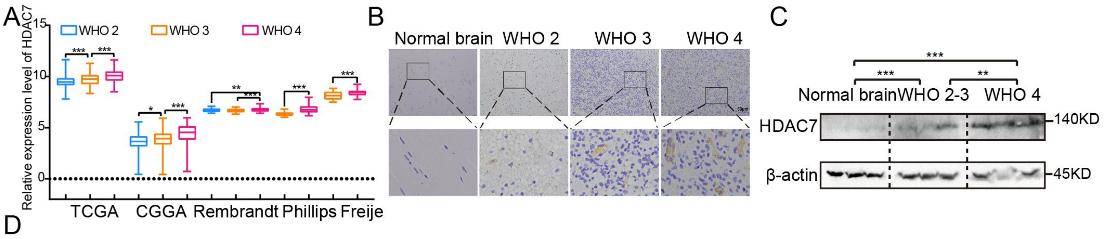

D

Genes	TCGA-GBM		CGGA-GBM		Gravendeel-GBM	
	Log-rank p value	Wilcoxon p value	Log-rank p value	Wilcoxon p value	Log-rank p value	Wilcoxon p value
HDAC1	0.1212	0.342	0.0391	0.1101	0.4589	0.3696
HDAC2	0.1309	0.0482	0.3622	0.7952	0.7212	0.3755
HDAC3	0.9538	0.6236	0.9725	0.8690	0.1680	0.0950
HDAC4	0.0901	0.263	0.7544	0.5233	0.7765	0.7445
HDAC5	0.8426	0.9501	0.0023	0.0017	0.1116	0.2575
HDAC6	0.5359	0.8511	0.1024	0.0506	0.5003	0.8715
HDAC7	0.0037	0.0033	0.0091	0.0788	0.0437	0.0273
HDAC8	0.6614	0.6876	0.4675	0.9631	0.0877	0.3188
HDAC9	0.9862	0.8048	0.4386	0.9774	0.2155	0.3254
HDAC10	0.2108	0.0753	0.3638	0.5979	0.7276	0.5066
HDAC11	0.4534	0.3962	0.2191	0.4545	0.5080	0.6869

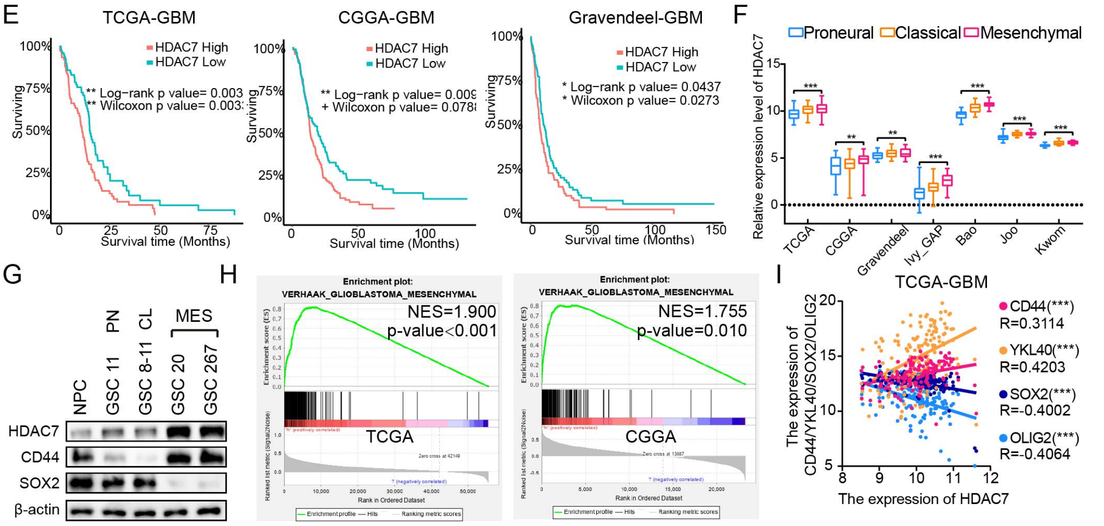

Supplementary Figure 2. HDAC7 suggests a poor prognosis, and its expression level increases with glioma grade.

A Expression levels of HDAC7 in different WHO grade of glioma in TCGA, CGGA, Gravendeel, Phillips and Freije datasets. **B** Representative IHC-staining images showing the relative expression of HDAC7 in normal brain and different WHO grade glioma samples. Scale bar, 50 μm. **C** Western blot showing the protein expression of HDAC7 in normal brain and different WHO grade glioma samples. **D** Significance analysis of survival curves for HDAC family genes in TCGA-GBM, CGGA-GBM and Gravendeel-GBM datasets. **E** Kaplan-Meier curves showed overall survival analyses for low and high expression HDAC7 in TCGA-GBM, CGGA-GBM and Gravendeel-GBM datasets. **F** Expression levels of HDAC7 in proneural, classical and mesenchymal phenotype glioma from the TCGA, CGGA, Gravendeel, Ivy-GAP, Bao, Joo and Kwom datasets. **G** Western blot showing the protein expression of HDAC7，CD44 and SOX2 in NPC (neural progenitor cell), PN (GSC 8-11), CL (GSC 11) and MES (GSC 20 and GSC 267) phenotype cell. **H** GSEA revealed that HDAC7 expression is positively enriched in GBM MES phenotype. **I** Correlation analysis of HDAC7 expression level with MES phenotype markers

(CD44 and YKL40) and PN phenotype markers (SOX2 and OLIG2) in TCGA-GBM dataset. All data are presented as the means ± SEM. The statistical significance is shown as * $P < 0.05$; ** $P < 0.01$; *** $P < 0.001$.

Supplementary Figure 3

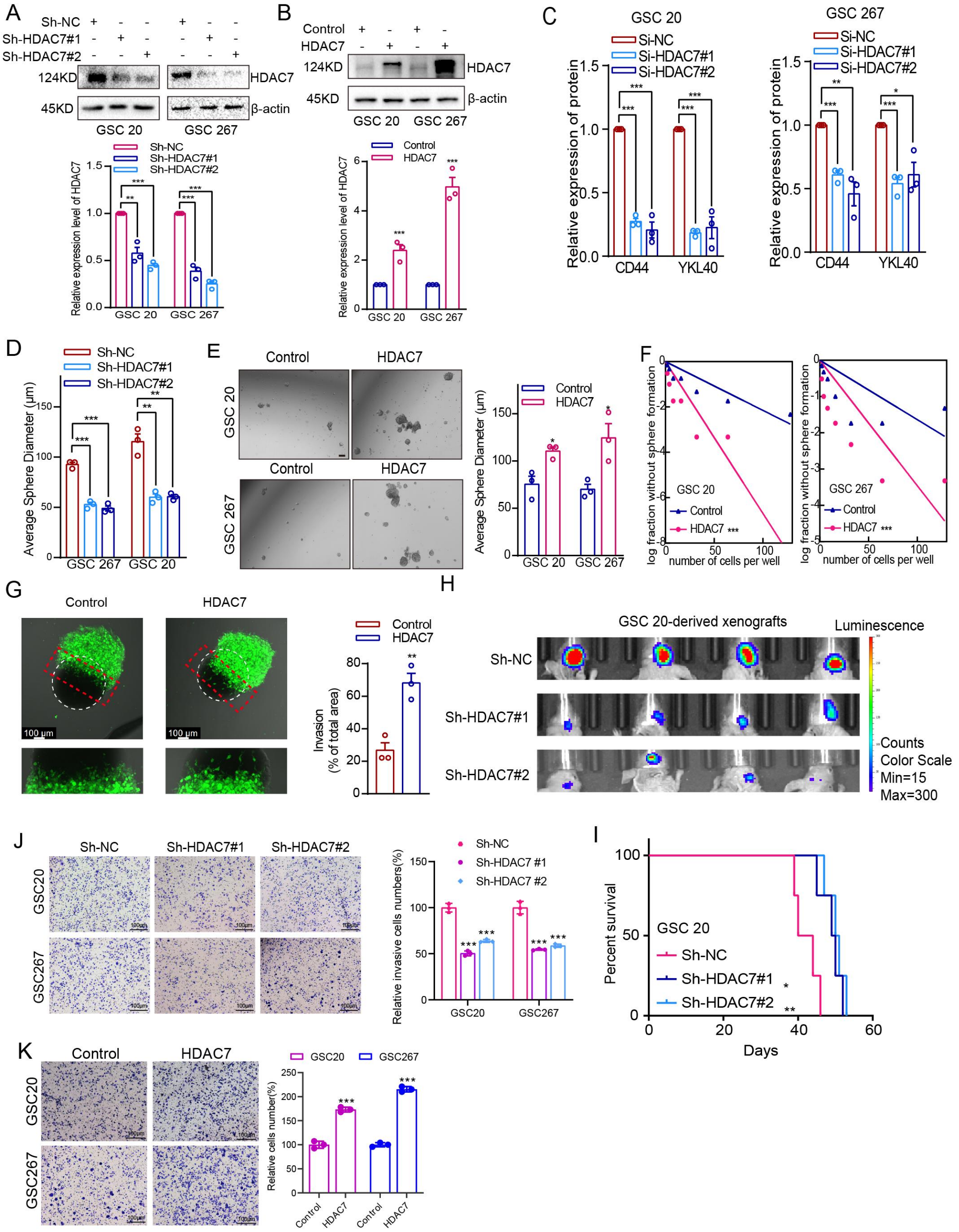

Supplementary Figure 3. HDAC7 promotes self-renewal and malignant phenotypes of GSCs in vitro and in vivo.

Representative western blot assays showing the protein expression level of HDAC7 in (**A**) HDAC7-knockdown and (**B**) HDAC7-overexpression GSC 20 and GSC 267. Quantification histogram showing the protein expression of HDAC7. **C** Quantification histogram showing the

protein expression of CD44 and YKL40 in HDAC7-knockdown GSC 20 and GSC 267. **D** The diameters of the tumour spheres of HDAC7-knockdown GSC 20 and GSC 267 were quantified. **E** The diameters of the tumour spheres of HDAC7-overexpression GSC 20 and GSC 267 were photographed and quantified. Scale bar, 100 μm. (**F**) limiting dilution assay, and (**G**) ex vivo co-culture invasion assays for GSCs transfected with ov-NC or ov-HDAC7. **H** Bioluminescence images showing the tumor size of mice implanted with luciferase-labelled GSC20 cells expressing sh-HDAC7 or sh-NC. **I** Kaplan-Meier survival curves for mice implanted with luciferase-labelled GSC20 cells expressing sh-HDAC7 or sh-NC. Log-rank analysis was used. **J** Representative images of THP-1 treated with PMA (100 ng/mL) migration after co-cultured with GSCs transfected with sh-NC, sh-HDAC7#1 and sh-HDAC7#2. scale bar, 100 μm. **K** Representative images of THP-1 treated with PMA (100 ng/mL) migration after co-cultured with GSCs transfected with Control or HDAC7. scale bar, 100 μm. All data are presented as the means ± SEM. The statistical significance is shown as * $P < 0.05$; ** $P < 0.01$; *** $P < 0.001$.

Supplementary Figure 4

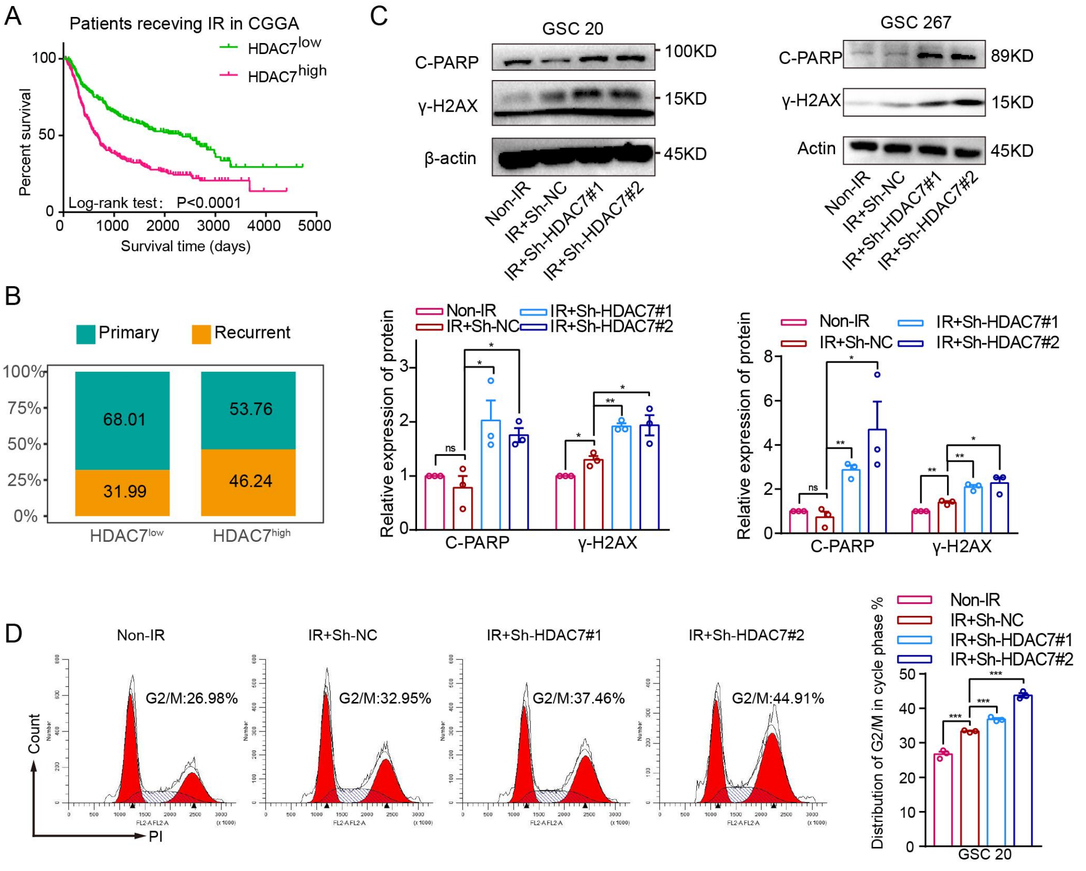

Supplementary Figure 4. HDAC7 knockdown enhances radiotherapy sensitivity of GSCs.

A Kaplan-Meier curves showed overall survival analyses for low and high expression HDAC7 in CGGA patients receiving IR. **B** Analysis of the proportion of primary and recurrent tumours in high and low HDAC7 expression subgroups from CGGA dataset. **C** Representative western blot assays showing the protein expression level of γ-H2AX and C-PARP in GSC20 and GSC267 after 6 Gy radiation only or combined with HDAC7 knockdown pretreatment. Quantification histogram showing the protein expression of γ-H2AX and C-PARP. **D** Cell cycle analysis of GSC20 after 6 Gy radiation only or combined with HDAC7 knockdown pretreatment.

All data are presented as the means ± SEM. The statistical significance is shown as * $P < 0.05$; ** $P < 0.01$; *** $P < 0.001$.

Supplementary Figure 5

A

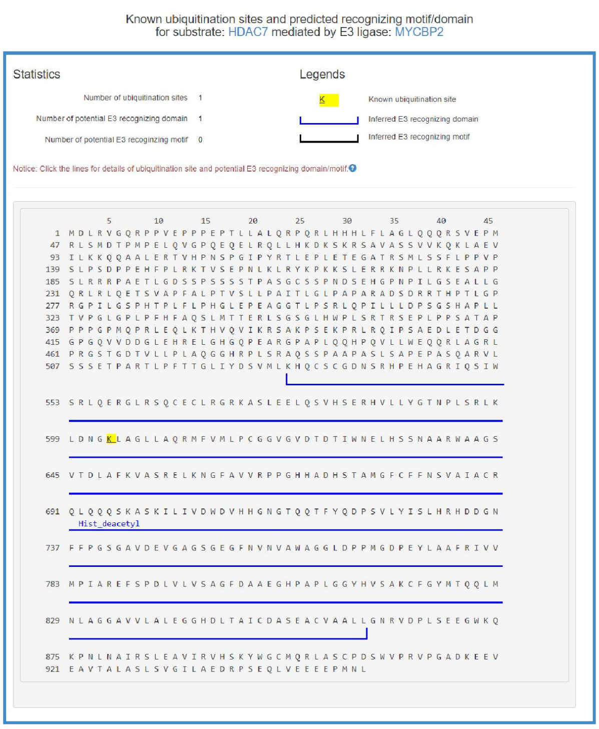

Supplementary Figure 5. MYCBP2 functions as an E3 ubiquitin-linked enzyme of HDAC7.

A UbiBrowser (http://ubibrowser.bioit.cn/ubibrowser/home/) is a ubiquitination prediction website that predicts the K603 site of HDAC7 is a potential ubiquitination site that is mediated by E3 ligase MYCBP2.

Supplementary Figure 6

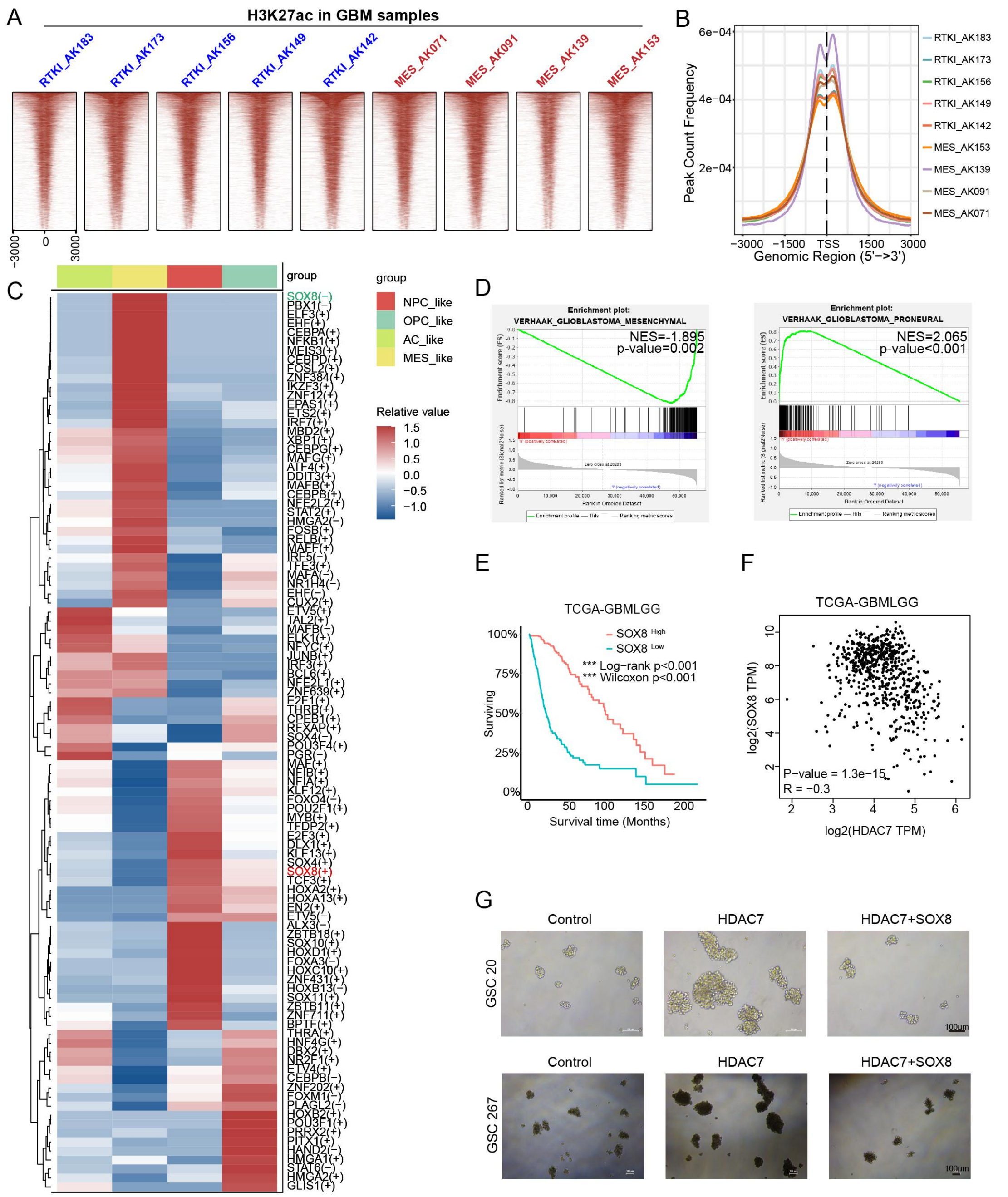

Supplementary Figure 6. SOX8 inhibits self-renewal and MES transformation of GSCs.

(A) Enriched heatmap visualisation of genome regions with differential chromosome accessibility, and **(B)** genome-wide profiles in five clinical RTK I and four MES GBM specimens, as identified by CHIP-seq analysis. **C** Heatmap of enriched TFs in NPC-like, OPC-like, AC-like and MES-like GBM. **D** GSEA analysis revealed that high SOX8 is negatively enriched in MES phenotype and positively enriched in PN phenotype. **E** Kaplan-Meier showed overall survival analyses for low and high expression SOX8 in TCGA dataset. **F** Correlation analysis of HDAC7 expression levels with SOX8 in TCGA-GBM datasets. **G** The tumour spheres for (**left**) GSC 20 and (**right**) GSC 267 transfected with ov-NC or ov-HDAC7 and ov-SOX8 as indicated were photographed. All data are presented as the means ± SEM. The statistical significance is shown as * $P < 0.05$; ** $P < 0.01$; *** $P < 0.001$.

Supplementary Figure 7

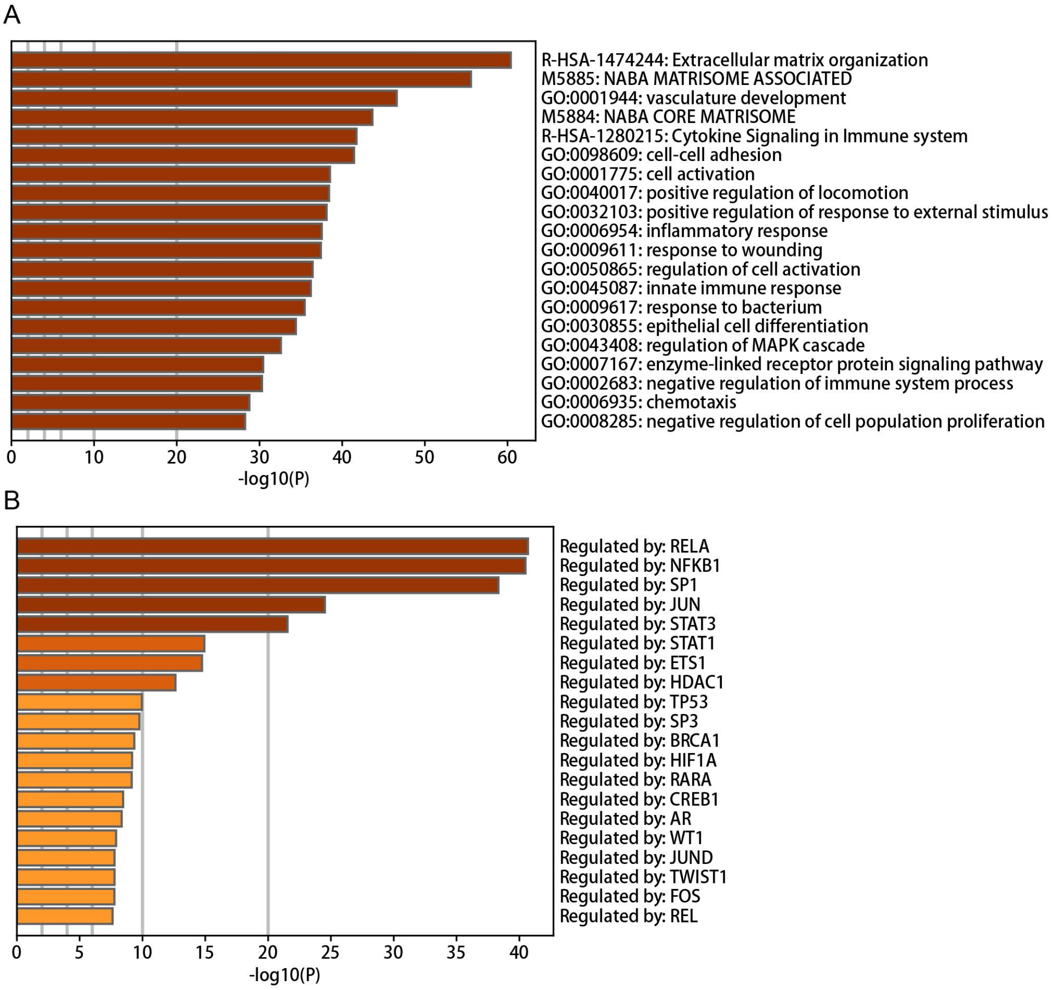

Supplementary Figure 7. Enrichment analysis of genes upregulated by HDAC7.

A GO functional enrichment analysis of genes upregulated by HDAC7. **B** An enrichment analysis in TRRUST was performed on genes that were upregulated by HDAC7, which demonstrated that the majority of these genes were regulated by the transcription factor JUN.

Supplementary Figure 8

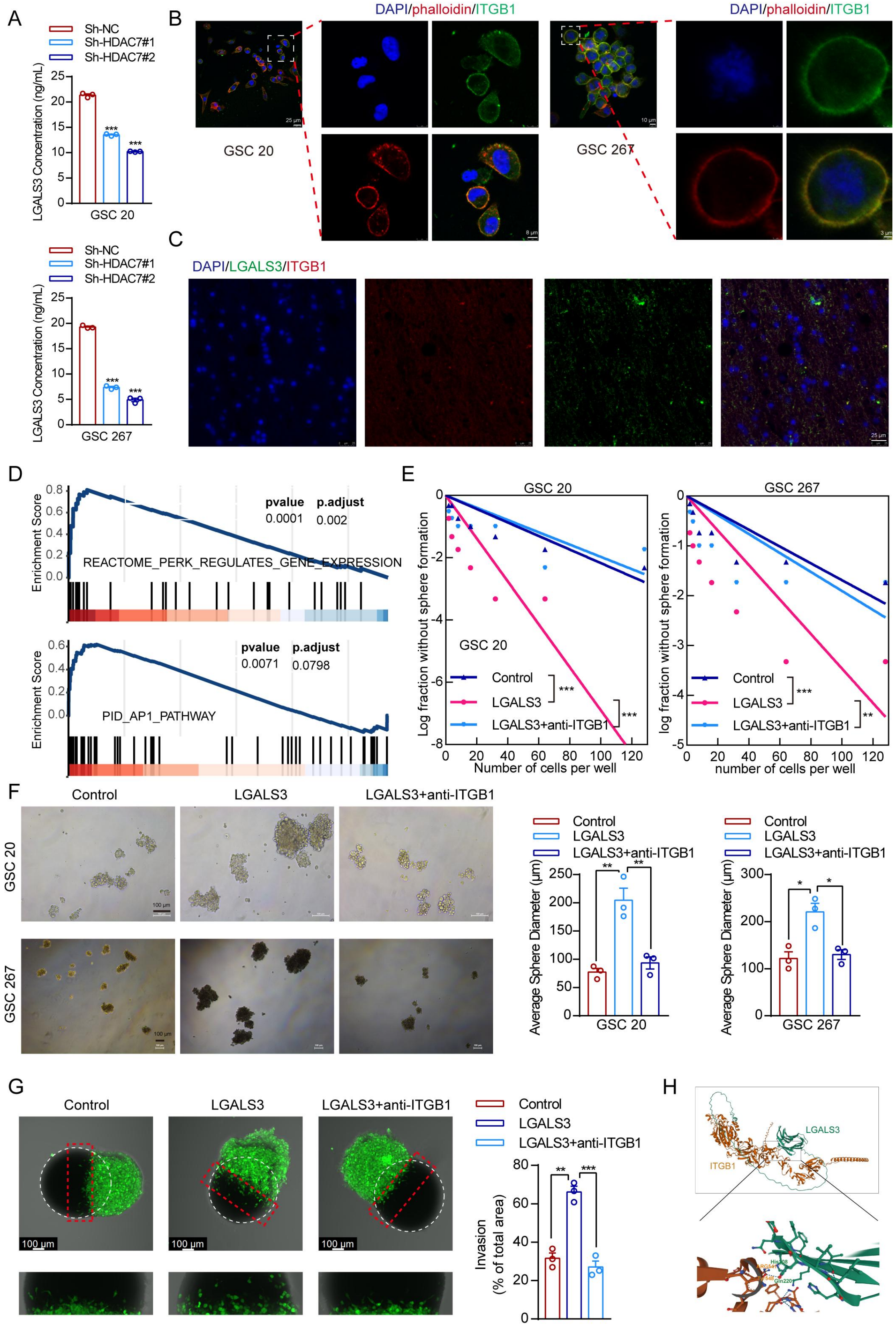

Supplementary Figure 8. LGALS3-ITGB1 mediates self-renewal and MES transformation of GSCs.

A ELISA was used to quantify the expression of the secreted protein LGALS3 produced by

GSCs. **B** Immunofluorescence staining experiments showing the colocalisation of phalloidin (red) and ITGB1 (green) in GSCs, revealing that ITGB1 is mainly present in the cell membrane. Scale bar is as shown. **C** Immunofluorescence staining experiments showing the colocalisation of ITGB1 (red) and LGALS3 (green) in GBM tissue. Scale bar, 25 μm. **D** GSEA analysis indicating that MES-like cells were significantly enriched in ERK and AP1 signaling pathway. (**E**) The diameters of the tumour spheres, (**F**) limiting dilution assay, and (**G**) ex vivo co-culture invasion assays for GSCs treated with recombinant protein LGALS3 or antibody ITGB1 as indicated. **H** The molecular docking model between LGALS3 and ITGB1 exhibiting 2 pairs of interacting amino acid residues (ARG541-His208,Tyr548-GLN220) maintained by hydrogen bonds, visualized by the VS Code software. Scale bar is as shown. All data are presented as the means ± SEM. The statistical significance is shown as * $P < 0.05$; ** $P < 0.01$; *** $P < 0.001$.

Supplementary Figure 9

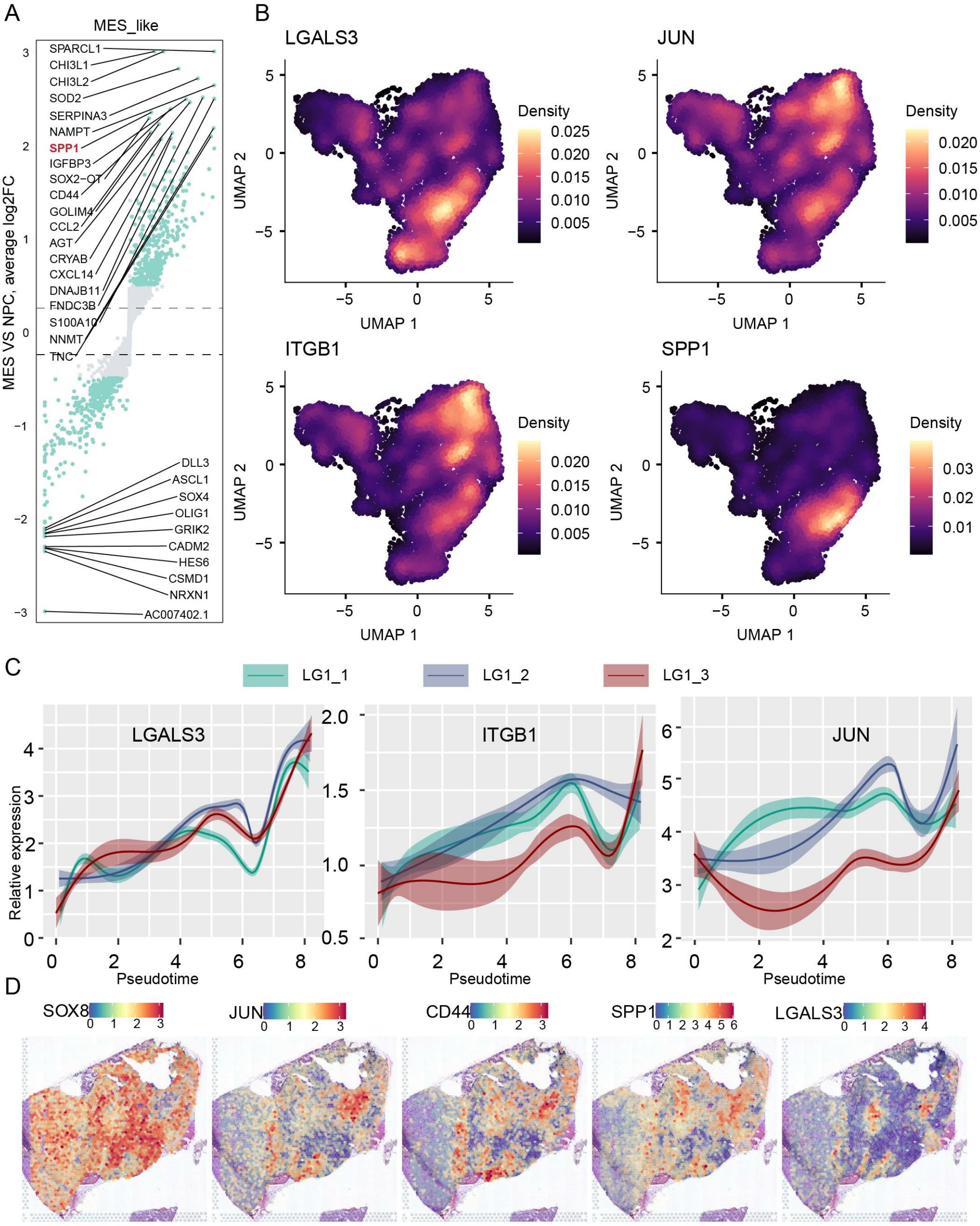

Supplementary Figure 9. HDAC7 promote macrophage infiltration via the LGALS3-ITGB1 axis.

A Volcano plot showing differential genes between MES and NPC. Padj was performed using Bonferroni correction. **B** Umap and density plots showing LGALS3, JUN, ITGB1 and SPP1 were highly expressed in the MES GBM subpopulation. **C** Monocle2 pseudotime analysis revealing that the expression of LGALS3, ITGB1 and JUN was gradually upregulated with tumour progression. **D** Spatial transcriptome data demonstrating the spatial exclusivity of SOX8 expression with the expression of JUN, CD44, SPP1 and LGALS3.

Supplementary Figure 10

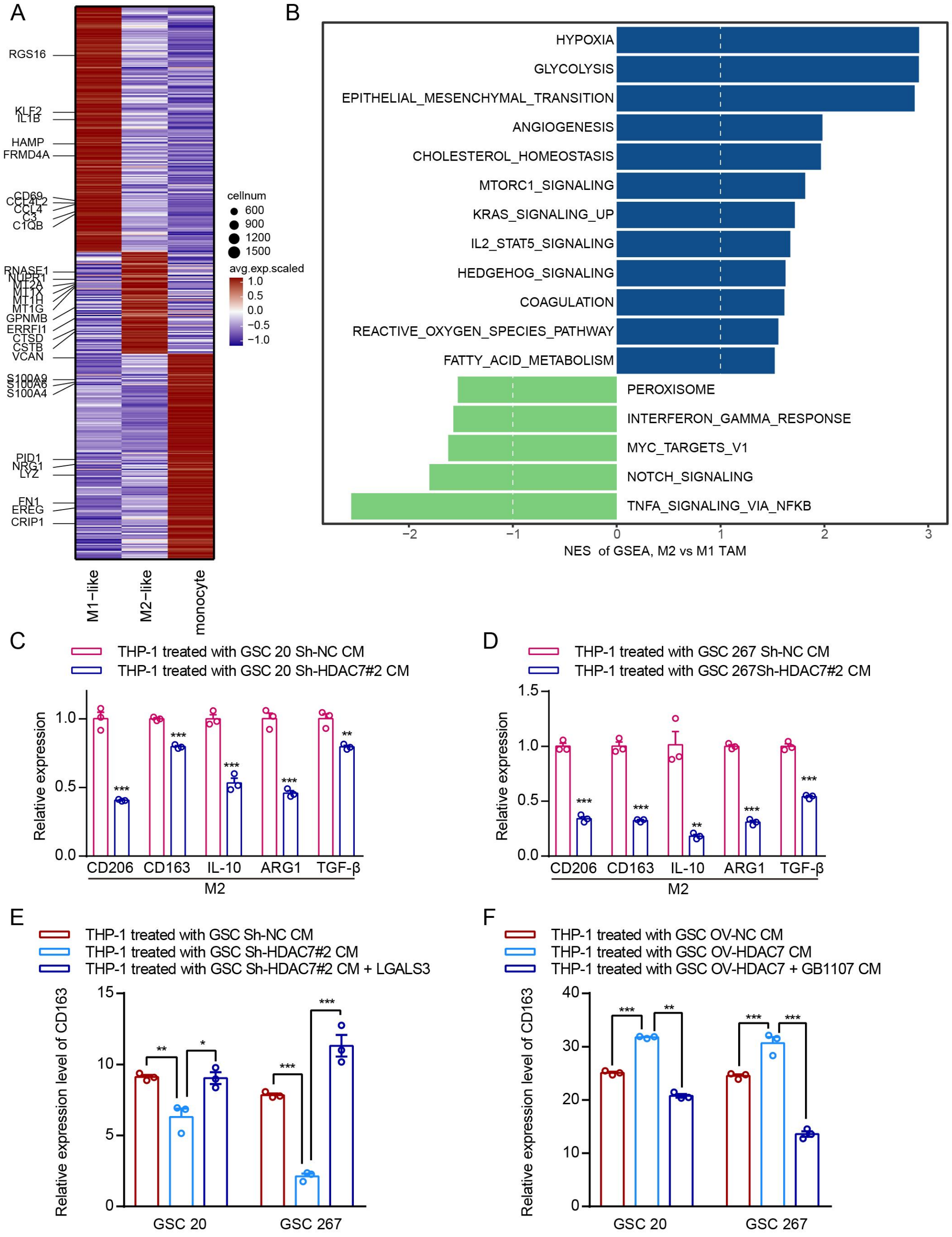

Supplementary Figure 10. LGALS3 secreted by GSCs promotes M2 polarization of TAMs in a paracrine manner.

A MDMs were annotated into three subpopulations (monocytes, M1-like and M2-like subpopulations) based on marker genes. **B** GSVA functional enrichment analysis revealing that M2-like macrophages were mainly enriched in tumour-promoting pathways. Expression levels of M2 marker mRNA detected by RT-qPCR in THP-1 treated with (**C**) GSC 20 or (**D**) GSC 267 Sh-NC CM or Sh-HDAC7#2 CM. **E** Statistical analysis of CD163 on THP-1 macrophages treated

with GSCs Sh-NC CM, Sh-HDAC7#2 CM or Sh-HDAC7#2 CM supplemented with recombinant protein LGALS3. **F** Statistical analysis of CD163 on THP-1 macrophages treated with GSCs OV-NC CM, OV-HDAC7 CM or OV-HDAC7 supplemented with GB1107 CM. All data are presented as the means ± SEM. The statistical significance is shown as * $P < 0.05$; ** $P < 0.01$; *** $P < 0.001$.

Supplementary Materials and Methods

Human glioma specimens and glioma stem cells

All patient-derived glioma stem cell (GSC) lines and neural progenitor cells (NPCs) were generously donated by dr. Krishna P.L. Bhat (The University of Texas, M.D. Anderson Cancer center, Houston, TX). GSC lines (GSC8-11, GSC8-11, GSC20, GSC267) and NPCs have been established and used extensively in previous studies. GSCs were cultured in DMEM/F12 (Gibco, USA) supplemented with 2% B-27 serum-free supplement (Gibco, USA), 20 ng/mL human recombinant EGF (R&D Systems, USA) and 20 ng/mL human recombinant bFGF (R&D Systems, USA). All GSCs and NPCs were incubated at 37 °C and 5% CO_2. Accutase solution (Sigma-Aldrich, USA) was utilized to digest the tumour spheroids. All cells were determined to be free of mycoplasma contamination prior to the beginning of this study.

Tissue dissociation, cDNA synthesis and single cell RNA-Seq library preparation

Collect samples according to the experimental design (PBMCs, Cell lines, tissues). Tissue were washed twice with phosphate buffer saline (PBS). The tissue was cut into small pieces about 1 mm^3 in size and placed in petri dish with appropriate amount of PBS, then transferred to centrifuge tube, adding appropriate amount of enzyme and shaking at a certain temperature for a period of time; After 2-3 minutes' standing, take the supernatant, use a filter membrane to remove large clumps; Following centrifuge the cell and the supernatant was decanted and discarded, resuspended cells with red blood cell lysis buffer and incubation 2-3 min at room temperature and then centrifuge (120×g, 4°C, 3min), samples were lastly resuspended with PBS. Then, cell suspensions (300-600 living cells per microliter determined by Count Star) were loaded on a Chromium Single Cell Controller (10x Genomics) to generate single-cell gel beads in emulsion (GEMs) by using Single Cell 3' Library and Gel Bead Kit V2 (10x Genomics, 120237) and Chromium Single Cell A Chip Kit (10x Genomics ,120236) according to the manufacturer's protocol. Single-cell RNA-seq libraries were prepared using Single Cell 3' Library Gel Bead Kit V2 following the manufacture's *introductio*n. Finally, sequencing was performed on an Illumina Novaseq6000 with a sequencing depth of at least 100,000 reads per cell and pair end 150bp (PE150).

Single cell data preprocess

Raw FASTQ files were mapped to the Reference genome (GRCh38/hg38) using Cell Ranger 3.0 (10x Genomics). To create Cell Ranger-compatible reference genomes, the references were rebuilt according to instructions from 10x Genomics, which were performed alignment, filtering, barcode counting and UMI counting. Following alignment, digital gene expression (DGE) matrices were generated for each sample and for all samples. Merged 10x Genomics DGE files were generated using the aggregation function of the Cell Ranger pipeline. All the cells in different batches

were merged together normalized by equalizing the read depth among libraries. The final result was the matrix of all cells and their global gene expressions.

UMAP visualization and determination of the major cell types

Gene expression analysis and cell type identification was analysed using Seurat V2.0 pipeline (http://satijalab.org/seurat/) after filtering and normalization, another R toolkit for single-cell transcriptomics (Butler et al., 2018). As the data were already normalized, they were loaded into Seurat without normalization, scaling or centring. Along with the expression data, metadata for each cell was collected, including information location. Next, highly variable genes were identified and used as input for dimensionality reduction via principal component analysis (PCA). The resulting PCs and the correlated genes were examined to determine the number of components to include in downstream analysis. UMAP was then performed on the first 20 principal components to visualize cells in a two-dimensional space. To identify differentially expressed genes in each cluster, the Seurat function FindAllMarkers was used. For a gene to be differentially expressed in a cluster it must be expressed by at least 10% of cells, have a log-fold change greater than 0.25, and reach statistical significance of an adjusted $p < 0.05$ as determined by the Wilcox test. Finally, cell clusters were annotated to known biological cell types using canonical marker.

Pseudotime Analysis

Single cell trajectory was analyzed using matrix of cells and gene expressions by Monocle 2. Differentially expressed genes or significantly variable genes among cells were identified and used for dynamic trajectory analysis which ordered cells in pseudotime. First, the expression of transcripts of each gene was determined. Genes were then ranked using the coefficient of variation versus mean metric, selecting the top 20 genes for each celltype as features. The resulting velocity estimates were projected onto the UMAP embedding obtained in Seurat.

Functional analysis of differential genes

We performed differential gene expression analysis of all cell types using Wilcoxon rank sum test inbuilt in Seurat package. Next, to assign pathway activity estimates to individual cells, we applied GSVA using standard settings, as implemented in the GSVA package (version 1.22.4)[1]. GSEA was performed by using GSEA software version 4.0.3[2], which uses predefined gene sets from the Molecular Signatures Database (MSigDB v7.1)[3].

Transcription factor regulon analysis

The analysis of the regulatory network and regulon activity was performed by pySCENIC[4]. The regulon activity (measured in AUC) was analyzed by AUCell module of the pySCENIC, and the active regulons were determined by AUCell

default threshold. The differential-expression regulon was identified by Wilcoxon rank-sum test in “FindAllMarkers” function in R package Seurat with following parameters: min.pct = 0.1, logfc.threshold = 0.25, pseudocount.use = F, only.pos = T. The scaled expression of regulon activity was used to generate a heatmap. The rank diagram shows the specific TF enriched with different subtypes[5].

Spatial transcriptomics data processing

The spatial transcriptomic data of GBM were downloaded from GEO database, and analyzed in R using the Seurat 4.0 package according to the recommended data processing guidelines (https://satijalab.org/seurat/articles/spatial_vignette.html). In briefly, The SCTranform function was used for data normalization, followed by PCA and UMAP for dimension reduction, and clustering was conducted with the default resolution of the first 20 PCs. The gene expression features were visualized by SpatialFeaturePlot function, and signature scoring (Table S1) for MES-like, AC-like, NPC-like and OPC-like malignant cells and MDMs was performed with the AddModuleScore function.

Molecular Docking

The molecular docking between ITGB1 (UniProt ID:P05556) and LGALS3(UniProt ID: P17931) was carried out via the AlphaFold Server (https://alphafoldserver.com/)[6], of which the results were visualized by the Visual Studio Code.

In vitro limiting dilution assays and tumorsphere formation assays

For the in vivo limiting dilution assay, GSCs were cultured in 96-well plates at a density gradient of 0, 2, 4, 8, 16, 32, 64 and 128 cell per well, with 10 replicates of each density gradient. After 7 days of cell culture, the number of wells forming tumour spheres was counted using inverted microscopy. For tumorsphere formation assays, we added 1000 GSCs to each well of 6-well plates. On the 7th day after the seeding of the cells, we counted the number of tumorspheres utilizing an inverted microscope.

Macrophage infiltration assays

The Macrophage infiltration assays were used to assess the chemotactic capabilities in different transfected GSC cells. In brief, different transfected GSC cells were inoculated into a 24-well plate. Then, THP1 cells were in turn centrifuged, resuspended in the serum-free medium, and counted by the automated cell counter. After adjusting the cell concentration, 100 μL of cell suspension containing 3–5 × 104 (For migration) or 5–7 × 104 (For invasion) cells was inoculated into the Transwell chambers (BIOFIL, China) coated (For invasion) or not (For migration) with the Matrigel matrix (Corning, USA), which were then placed into a 24-well plate containing 700 μL of complete medium. Following 24 h of incubation at 37 °C, the cells were fixed with methanol for 15 min and then stained with 0.5% of crystal violet for 30 min. Finally, the cells crossing the basal membrane of the Transwell chamber

were counted with the Image J software.

Cycloheximide (CHX) and MG132 treatment

For cycloheximide (CHX, Selleck, china) treatment, GSC cells were respectively cultured with medium containing 25μg/mL CHX for indicated time. The extracted proteins were subjected western blotting. For MG132 (MedChemExpress) treatment, GSC cells were cultured with medium containing 10μM MG132 for 12 hours. The extracted proteins were subjected western blotting.

Ionizing radiation treatment

For in vitro experiments, GSCs in 6-well plates were irradiated at a dose of 6 Gy and incubated in an incubator for 48 hours for subsequent experiments. For animal experiments, mice were anaesthetized using a gas anaesthesia system and 2.5 Gy of radiation was applied for 4 consecutive days. All ionizing radiation treatments were performed at the Department of Radiation Oncology, Qilu Hospital, Shandong University.

Immunoblotting, immunohistochemistry, and immunofluorescence:

For immunoblotting, GSCs were lysed using RIPA buffer containing 1% protease and phosphate inhibitor cocktail (P8340; Sigma-Aldrich, USA). The supernatant was subsequently obtained by centrifugation at 13000 g and 4 °C for 10 minutes. Subsequently, the supernatant fraction was transferred to a PVDF membrane after SDS-PAGE. The PVDF membrane was blocked with 5% skimmed milk for 1 hour. The strips were then incubated with primary antibody at 4 °C overnight and detected with secondary antibody. For immunohistochemistry, surgical specimens from glioma patients and intracranial tumours from nude mice were embedded in paraffin. The immunohistochemistry steps were performed as described previously. For immunofluorescence, GSCs were fixed in 4% paraformaldehyde, permeabilized with 0.5% Triton X-100 and blocked with goat serum for 1 hour at room temperature. Next, the indicated primary antibodies were incubated with GSCs at 4 °C overnight. The next day, GSCs were washed with PBS and incubated with the corresponding Alexa Fluor 488 or Alexa Fluor 594 secondary antibodies and DAPI. Finally, GSCs were observed with the assistance of a confocal laser scanning microscope. A complete list of antibodies can be found in Table S6.

Cell cycle analysis

GSCs were treated by fixation in precooled 70% ethanol. After ethanol fixation, GSCs were incubated with propidium iodide and RNase A. Finally, flow cytometry was used to perform cell counting.

Apoptosis assay

The FITC-Annexin V Apoptosis Detection Kit (BD Biosciences, USA) was used to detect the proportion of apoptotic cells according to the manufacturer's protocol.

GSCs were collected and analysed by a BD Accuri C6 flow cytometer (BD Biosciences, USA).

Enzyme-linked immunosorbent assay (ELISA)

The Human LGALS3 ELISA kit was purchased from Proteintech (KE00126). All procedures were performed according to the manufacturer's instructions.

Immunoprecipitation

GSCs with the appropriate treatment were collected and lysed with immunoprecipitation lysis solution. Cell lysates were incubated with protein A/G magnetic beads and the indicated antibodies. Finally, the magnetic beads were washed three times using immunoprecipitation buffer. The bound proteins were collected and then analysed by immunoblotting.

RNA extraction and real-time quantitative PCR (RT–qPCR)

Total RNA was extracted from GSCs using TRIzol reagent (Invitrogen, USA) and reverse transcribed according to the instructions of the RNA Reverse Transcription kit (TaKaRa, Japan). The expression levels of the target genes were determined using the $2^{-\Delta\Delta Ct}$ method and normalized to the gene β-actin. Oligonucleotide primers for the corresponding target genes are presented in Table S7.

Chromatin immunoprecipitation assay

The final concentration of 1% formaldehyde was added to the GSC medium at 37 °C for 10 minutes. Cell lysate was used to lyse the crosslinked GSCs. Ultrasonication sheared the crosslinked chromatin to a size of 100-300 bases. The chromatin supernatant separated by centrifugation was incubated with anti-H3K27ac antibody, anti-JUN antibody or anti-IgG antibody overnight at 4 °C with rotation. The DNA obtained by elution was purified and quantified using RT-qPCR.

Animal studies

Four-week-old male BALB/c nude mice were purchased from SLAC Laboratory Animal Centre (Shanghai, China). All nude mice were raised under pathogen-free conditions. For the intracranial in situ tumour model, we injected lentiviral-transfected luciferase-expressing GSCs (5×10^5) stereotactically into the right frontal lobe of each mouse. Mice in the treatment groups were injected intraperitoneally with SAHA (20 mg/kg) or orally with GB1107 (20 mg/kg) for 14 consecutive days. The kinetics of tumour growth were monitored by bioluminescence imaging using the IVIS Lumina series III ex vivo imaging system (PerkinElmer, USA) and quantified by in vivo imaging software. Meanwhile, the survival time of the mice was recorded.

References

1. Hänzelmann S, Castelo R, Guinney J. GSVA: gene set variation analysis for microarray and RNA-seq data. BMC Bioinformatics. 2013; 14: 7.

2. Subramanian A, Tamayo P, Mootha VK, Mukherjee S, Ebert BL, Gillette MA, et al. Gene set enrichment analysis: a knowledge-based approach for interpreting genome-wide expression profiles. Proc Natl Acad Sci U S A. 2005; 102: 15545-50.
3. Liberzon A, Birger C, Thorvaldsdóttir H, Ghandi M, Mesirov JP, Tamayo P. The Molecular Signatures Database (MSigDB) hallmark gene set collection. Cell Syst. 2015; 1: 417-25.
4. Aibar S, González-Blas CB, Moerman T, Huynh-Thu VA, Imrichova H, Hulselmans G, et al. SCENIC: single-cell regulatory network inference and clustering. Nat Methods. 2017; 14: 1083-6.
5. Neftel C, Laffy J, Filbin MG, Hara T, Shore ME, Rahme GJ, et al. An Integrative Model of Cellular States, Plasticity, and Genetics for Glioblastoma. Cell. 2019; 178: 835-49.e21.
6. Abramson J, Adler J, Dunger J, Evans R, Green T, Pritzel A, et al. Accurate structure prediction of biomolecular interactions with AlphaFold 3. Nature. 2024; 630: 493-500.

Supplementary Table 6-7

Table S6-Antibodies used in this study	
**Name**	**Resources**
**Antibodies for immunoblotting**	
β-actin	Cell Signaling Technology, 8480
HDAC7	Cell Signaling Technology, 10831/33418<br>Santa cruz, sc-74563
C\EBPβ	Cell Signaling Technology, 43095
YKL40	Cell Signaling Technology, 47066
CD44	Cell Signaling Technology, 37259
SOX2	Cell Signaling Technology, 23064
HA	Cell Signaling Technology, 3724
FLAG	Cell Signaling Technology, 14793
TRIM28	Santa cruz, sc-515790
SUMO3	Abcam, ab203570
Ubiquitin	Cell Signaling Technology, 3936
His	Cell Signaling Technology, 12698
MYCBP2	Abcam, ab86078
H3K27ac	Cell Signaling Technology, 8173
SOX8	Proteintech, 20627-1-AP
JUN	Cell Signaling Technology, 9165
ITGB1	Cell Signaling Technology, 9699
LGALS3	Cell Signaling Technology, 87985
P-FAK	Cell Signaling Technology, 8556
FAK	Cell Signaling Technology, 71433
P-ERK	Proteintech, 28733-1-AP
ERK	Proteintech, 11257-1-AP
P-JUN	Cell Signaling Technology, 3270
SPP1	Cell Signaling Technology, 88742
**Antibodies for immunohistochemistry**	
HDAC7	Cell Signaling Technology, 33418
ITGB1	Cell Signaling Technology, 9699
LGALS3	Cell Signaling Technology, 87985
SOX8	Proteintech, 20627-1-AP
CD44	Cell Signaling Technology, 37259
SPP1	Cell Signaling Technology, 88742
**Antibodies for immunofluorescence**	
HDAC7	Cell Signaling Technology, 10831<br>Santa cruz, sc-74563
TRIM28	Santa cruz, sc-515790
SUMO3	Abcam, ab203570
HA	Cell Signaling Technology, 3724
MYCBP2	Abcam, ab254951
SOX8	Proteintech, 20627-1-AP
JUN	Santa cruz, sc-74543
LGALS3	Cell Signaling Technology, 87985
ITGB1	Abcam, ab30394
**Antibodies for immunoprecipitation**	
HA	Cell Signaling Technology, 3724
FLAG	Cell Signaling Technology, 14793
LGALS3	Cell Signaling Technology, 87985

**Antibodies for chromatin immunoprecipitation (CHIP)**	
H3K27ac	Cell Signaling Technology, 8173
JUN	Cell Signaling Technology, 9165
**Antibodies for RNA immunoprecipitation (RIP)**	
HA	Cell Signaling Technology, 3724

Table. S7 Oligos and primers used in this study	
**Name**	**Sequence (5'>3')**
**Oligos for siRNA**	
Si-HDAC7	#1: AAGUAGUUGGAACCAGAGAA<br>#2: ACUGACCUCGCCUUCAAAGTT
Si-TRIM28	#1: UGGCUCUGUUCUCUGUCCUTT<br>#2: GAGACCAAACCUGUGCUUATT
Si-MYCBP2	#1: GGGCCAUAUAUACAAUUCUTT<br>#2: CCCGAGAUCUUGGGAAUAATT
**Primers for quantitative RT-PCR**	
β-actin	Forward:GAAGAGCTACGAGCTGCCTGA<br>Reverse:CAGACAGCACTGTGTTGGCG
HDAC7	Forward:TCCACTGCTCCGAAAGGAGA<br>Reverse:CTCGCTGTCATTGGGGGAG
SOX8	Forward:AGAAGGACCACCCCGACTAC<br>Reverse:AGCCCTGCTTCAGCCTTGTA
ITGB1	Forward:ATCTGCGAGTGTGGTGTCTG<br>Reverse:AAGGCTCTGCACTGAACACA
LGALS3	Forward:GGTGAAGCCCAATGCAAACA<br>Reverse:TGTTCTCATTGAAGCGTGGGT
CD206	Forward:GATTGCAGGGGGCTTATGGG<br>Reverse:CGGACATTTGGGTTCGGGAG
CD163	Forward:GGCTTGCAGTTTCCTCAAGA<br>Reverse:GACACAGAAATTAGTTCAGCAGCA
IL-10	Forward:GGCACCCAGTCTGAGAACAG<br>Reverse:TGGCAACCCAGGTAACCCTTA
ARG1	Forward:TGACGGACTGGACCCATCTT<br>Reverse:GGCTTGTGATTACCCTCCCG
TGF-β	Forward:TCCGTGGAGCTGAAGCAATA<br>Reverse:CTGCAAGTGGACATCAACGG
SOX8 Promoter	Forward:ACCTGGCTGCAGAGATGAGA<br>Reverse:GCCTTTGGAGGAAGCATTGG
ITGB1 Promoter a1	Forward:GTTTCCAGCCCCAATCTCCA<br>Reverse:TGAGTTGCGTCCTGCTTTTG
ITGB1 Promoter b1	Forward:GGCTCACGGTCCCGATTT<br>Reverse:GGCCCGTGTTCTTTAAGTTCC
ITGB1 Promoter c1	Forward:CGCAGAGGAGGAAACTGAGG<br>Reverse:CTAGGAGGAGGCGGAGGATG
LGALS3 Promoter a2	Forward:CATCCGTAGAAACCAGACAGC<br>Reverse:AAGCGTAAAAAGGAACCCAATAAA
LGALS3 Promoter b2	Forward:AATCAGGTGTAAGCAAGCCC<br>Reverse:AAGTAGATAGCCAAATGAATTGCTG
LGALS3 Promoter c2	Forward:TTATTACCAATGGCTAACCCTCTAA<br>Reverse:AAAAGGTATCACCCAAAGGAATGC
LGALS3 Promoter d2	Forward:CCTATTAATTGAATTCCTCTCAGCG<br>Reverse:CCACCGTTAGGTTCCGTGG
